# The potential of plant-derived vesicles in treating periodontitis and associated systemic diseases: current advances and future directions

**DOI:** 10.1186/s12951-025-03651-0

**Published:** 2025-08-18

**Authors:** Zongshuai Liu, Yonglin Guo, Yifei Deng, Juhua Shao, Xin Huang, Zhengguo Cao

**Affiliations:** 1 State Key Laboratory of Oral & Maxillofacial Reconstruction and Regeneration, Key Laboratory of Oral Biomedicine Ministry of Education, Hubei Key Laboratory of Stomatology, School & Hospital of Stomatology, Wuhan University, Wuhan, 430079 China; 2 Department of Periodontology, School & Hospital of Stomatology, Wuhan University, 237 Luoyu Road, Hongshan District, Wuhan, 430079 China

**Keywords:** Plant-derived vesicles, Periodontitis, Nomenclature, Systemic diseases, Oral microecology

## Abstract

**Background:**

Periodontitis, a chronic multifactorial inflammatory disease, represents a significant public health burden among global chronic non-communicable diseases. In addition to affecting oral health, periodontitis is closely associated with a variety of systemic diseases. Current treatments, including surgical and nonsurgical therapies, lack clear superiority, underscoring the need for innovative therapeutic strategies.

**Main body of the abstract:**

Plant-derived vesicles (PDVs), as natural products, have the advantages of being highly biocompatible, rich in biologically active components, and easy to cross biological barriers. Recent studies have shown that PDVs may treat periodontitis by maintaining oral microecological balance, remodeling the periodontal immune microenvironment, regulating inflammatory responses and oxidative stress, and promoting periodontal tissue regeneration. This review synthesizes the nomenclature based on MISEV 2023 and the latest research advances from biogenesis to removal, pre-processing, isolation, and characterization methods. It systematically evaluates potential applications of PDVs in periodontitis and associated systemic diseases, and presents the challenges facing current research.

**Conclusion:**

PDVs hold promise as a novel, multitargeted approach for periodontitis and its systemic systemic diseases. However, overcoming limitations in production consistency, mechanistic understanding, and regulatory frameworks is critical to advancing their clinical application. Future research should prioritize interdisciplinary collaboration to harness PDVs’ full therapeutic potential while addressing current translational barriers.

**Graphical Abstract:**

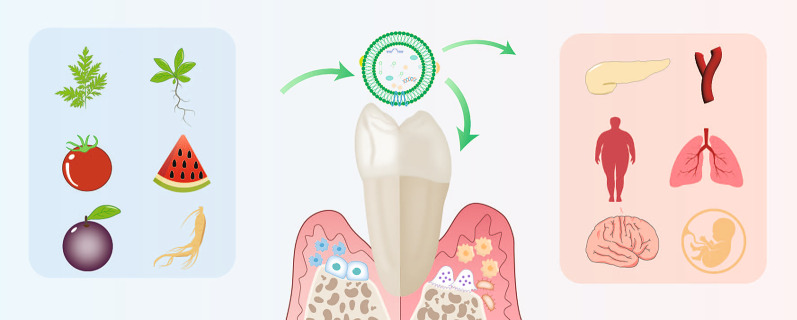

## Introduction

Periodontitis is a chronic multifactorial inflammatory disease characterized by pathologic loss of the periodontal supporting tissues [1, 2]. According to recent findings, the prevalence of periodontitis in dentate adults is 62%, while the prevalence of severe periodontitis is 23.6%. And its high prevalence increases the global burden of chronic non-communicable diseases as a public health challenge [3]. In addition to affecting oral health, periodontitis is also closely related to many systemic diseases, such as type II diabetes mellitus (T2DM), cardiovascular diseases, obesity, Alzheimer’s disease, and adverse pregnancy [4]. Therefore, treating periodontitis and focusing on associated systemic diseases are critical to maintaining patients’ general health. Treatments for periodontitis include both surgical and nonsurgical treatments, and no one periodontal treatment has shown clear superiority over any other periodontal treatments [5]. As a result, we have to face the limitations of today’s treatments, such as excessive tissue damage caused by laser therapy, and the inability of scaling and root planning to transform the infectious process into a homeostatic/commensal balance [6, 7].

**Fig. 1:**
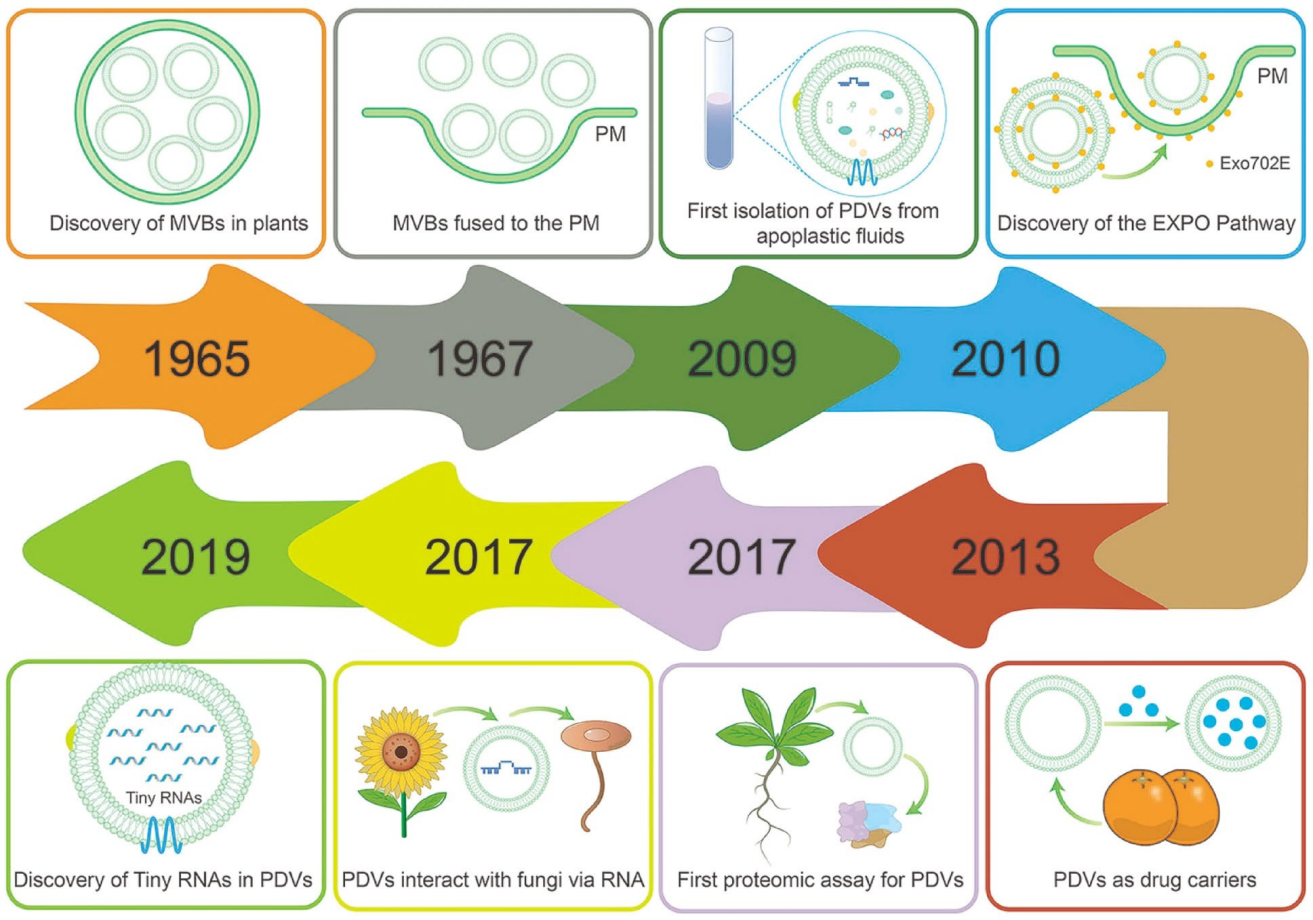
The timeline of PDVs research showcases significant years and noteworthy findings of PDVs

Extracellular vesicles (EVs) are defined as particles released from the cells, delimited by a lipid bilayer, and cannot replicate on their own [8]. Beginning in 1967, Halperin et al.. used transmission electron microscope (TEM) to observe and identify multivesicular vesicles (MVBs) that fuse with the plasma membrane and release exosome-like vesicles into the cell wall region of carrot cell cultures for the first time [9]. Since then, plant-derived vesicles (PDVs) have received much attention and entered a phase of rapid development (Fig. 1). As natural product derived PDVs, they have unique advantages over other derived vesicles, such as huge production, rich in bioactive materials, high biocompatibility and environmental friendliness [10]. Notably, previous studies have demonstrated that PDVs can readily pass through some biological barriers, such as the blood-brain barrier [11]. Therefore, many scholars consider PDVs as an emerging tool for disease treatment [12, 13].

In recent years, research has indicated that PDVs may play a role in the treatment of periodontitis and associated systemic illnesses. Sundaram et al.. showed that phosphatidic acid (PA) in ginger-derived vesicles (GG-DVs) inhibits the growth of *Porphyromonas gingivalis (P. gingivalis)*, the main pathogen of chronic periodontitis, which is expected to be used in the treatment of periodontitis [14]. Wang et al.. pioneering combination of the benefits of H_2_ therapy and PDVs for the treatment of T2DM. Using hollow mesoporous silica (HMS) nanoparticles to deliver ammonia borane with acid-corresponding H_2_-releasing capacity and covering it with GG-DVs to enhance the biocompatibility of HMS and modulate the intestinal flora. In this way, they solved the two main problems in treating T2DM: insulin resistance and pancreatic β-cell dysfunction [15]. It has also been found that garlic-derived vesicles (GL-DVs) reverse high-fat diet-induced obesity through the gut/brain axis [16]. Although still in development, there is no doubt that PDVs has yielded some encouraging findings in the treatment of periodontitis and associated systemic diseases.

In this review, this article will explore the prospects of PDVs in the treatment of periodontitis and associated systemic diseases, including the recent research advances in nomenclature based on MISEV 2023, from biogenesis to removal, pre-processing, isolation and characterization, highlighting the potential applications of PDVs in periodontitis and associated systemic diseases, and presents the challenges facing current research.

## Nomenclature

So far, the study of PDVs has given rise to a confusing array of terms, such as plant exosome nanovesicles (PENs) [17], plant-derived exosome-like nanoparticles (PDENs) [18], plant-derived vesicle-like nanoparticles (PDVLNs) [19], plant-derived nanovesicles (PDNVs) [20] and so on. These terms are characterized by the use of the terms “exosome” or “exosome-like” to imply a specific biogenesis pathway. Nevertheless, isolation of PDVs is not usually concentrated according to different biogenesis pathways, and these terms are misleading. Although some researchers have attempted to separate “extracellular vesicles” and “granules” by establishing protocols, the two terms are often confused in PDVs. And there is a lack of rigorous discussion to demonstrate the relationship between the two terms [21]. There have also been studies using microvesicles and nanovesicles, but there is no strict consensus on the upper and lower sizes [22]. As early as 2020, Pinedo *et al*. called for a standardization of PDVs nomenclature [23]. Unfortunately, no consensus has emerged to date.

**Fig. 2:**
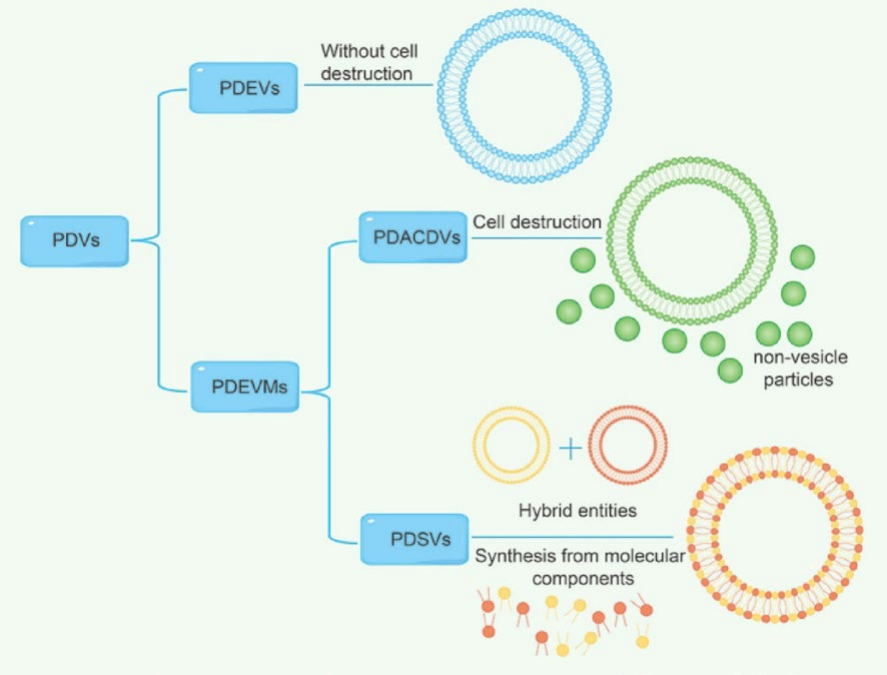
Nomenclature of PDVs based on MISEV 2023

The International Society for Extracellular Vesicles (ISEV) has issued minimal information for studies of extracellular vesicles (MISEV 2023) [8]. Therefore, we will use the new nomenclature for PDVs with reference to MISEV 2023 (Fig. 2).

According to MISEV 2023, we classify PDVs into plant-derived extracellular vesicles (PDEVs) and plant-derived extracellular vesicles mimetics (PDEVMs). The term “PDEVs” refers to particles that are released from plant cells, are delimited by a lipid bilayer, and cannot replicate on their own (i.e., do not contain a functional nucleus). PDEVs are usually pre-processed using tissue infiltration centrifugation. For example, Rutter *et al*. performed low-speed centrifugation on *Arabidopsis thaliana *rosettes using MES buffer vacuum infiltrated and packed in syringes, thus establishing the first study of isolating and purifying PDEVs from leaves [24]. The plant-derived extracellular vesicles mimetics (PDEVMs) can be used to denote EVs-like particles, which include plant-derived artificial cell-derived vesicles (PDACDVs) and plant-derived synthetic vesicles (PDSVs). The former refers to Evs mimetics that are produced in the laboratory under conditions of induced cell disruption, such as stirring. PDACDVs are usually pre-processed using tissue-disruption centrifugation. For instance, Cao *et al*. obtained ginseng-derived vesicles (GS-DVs) by grinding ginseng root into ginseng fluid in a slow juicer and centrifuging them [25]. In a number of previous studies, PDVs obtained by tissue-disruption centrifugation were often described as “nanoparticles”. The PDSVs refers to EVs mimetics that are synthesized de novo from molecular components or made as hybrid entities. Zhuang *et al*. fused of *Escherichia coli*-derived bacterial outer membrane vesicles and spinach-derived vesicles (SN-DVs) to construct in situ tumor vaccines with phytochemical features [26].

## From biogenesis to removal

### Biogenesis

**Fig. 3:**
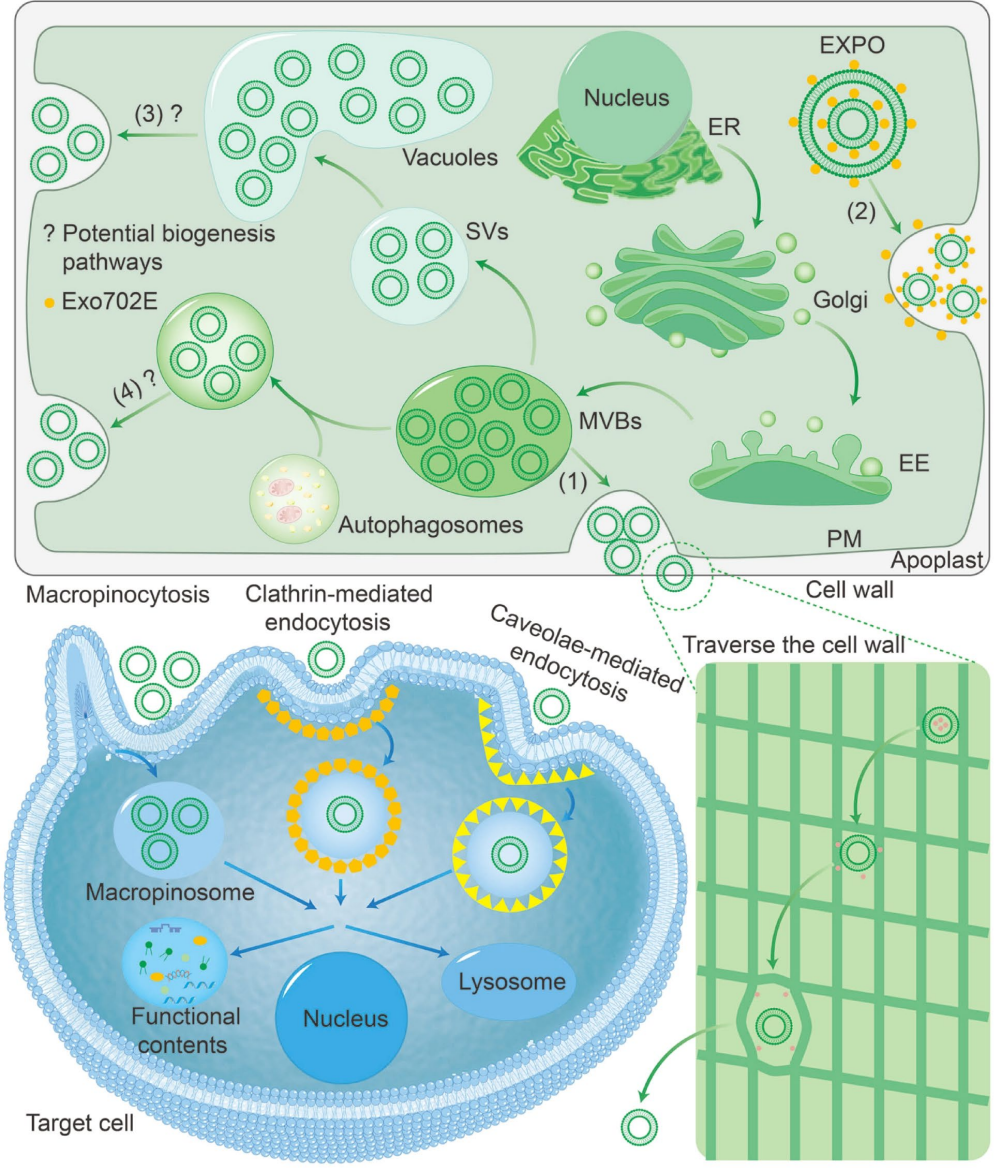
From biogenesis to removal of PDVs. Biogenesis of PDVs includes the MVBs pathway and the EXPO pathway, as well as potentially the vacuoles pathway and the autophagosomes pathway. PDVs disrupt and loosen the cell wall by releasing cell wall recombinant proteins. Target cells internalize PDVs via macropinocytosis, clathrin-mediated endocytosis, caveolae-mediated endocytosis. Some PDVs release functional contents to exert biological effects, and others are degraded by lysosomes.

So far, researchers have identified two biogenesis pathways for PDVs, including multivesicular bodies (MVBs) and exocyst-positive organelle (EXPO), in addition to some potential pathways suggested by researchers (Fig. 3).

#### MVBs pathway

MVBs are single membrane-bound organelles that contain multiple intraluminal vesicles (ILVs), hence the term multivesicular bodies [27]. MVBs fuse with the plasma membrane and release multiple vesicles into the extracellular environment [28]. The endosomal sorting complex required for transport (ESCRT) is the most important ubiquitin-dependent mechanism regulating the formation of MVBs and ILVs. It works by binding and sequestering ubiquitinated proteins and sorting them into the ILVs of the MVBs [29]. Recent studies have shown that transcriptional downregulation of the ESCRT gene will disrupt the biogenesis of PDVs [30].

#### EXPO pathway

In 2010, Wang et al.. identified EXPO for the first time in *Arabidopsis thaliana* and tobacco cells, mediating cytosol to cell wall exocytosis [31]. Researchers later defined EXPO as a specialized organelle representing an unconventional protein secretion pathway [32]. Specifically, EXPO fuses with the plasma membrane and releases individual membrane vesicles into the cell wall [31].

#### Other potential pathways

Many researchers believe that the vacuoles pathway is also involved in the biogenesis of PDVs [10, 33]. Specifically, MVBs fuse to produce small vacuoles (SVs), which act as nascent vesicles containing ILVs and continue to fuse to form larger vesicles [34]. Since the biogenesis of vacuoles does not arise solely from MVBs, two types of vacuoles were observed in the epidermal cells of grapefruit: empty vacuoles and vesicles-filled vacuoles [35]. It has also been found that large central vacuoles fuse with the plasma membrane in response to bacterial infection [36]. Unfortunately, no studies have directly observed the release of PDVs from vacuole. Fusion of autophagosomes with MVBs followed by fusion with the plasma membrane to release EVs has also been suggested as one of the possible pathways [37]. However, the autophagosomes pathway still lacks sufficient evidence in plant cells.

### Traverse the cell wall

One of the striking differences between the structure of plant and animal cells is that plant cells possess a cell wall composed of polysaccharides, proteins, and other biomolecules [38]. Physically, PDVs are much larger than the pore size of the cell wall, so how do they get through the cell wall to the target cell [39]? In a proteomic analysis of sunflower-derived vesicles, it was found that about 47% of the proteins were cell wall-associated proteins, including glycosyl hydrolases, expansins and arabinogalactan proteins, which are associated with cell wall reorganization [40]. Although the exact mechanism is unknown, it has been hypothesized that PDVs may be able to pass through the cell wall by disrupting the cell wall and loosening it via cell wall recombinant proteins [41]. Furthermore, bacterial EVs and fungal EVs that traverse their thick cell walls by turgor pressure or transcellular wall channels could then inform the study of PDVs [42].

### Internalization

Zhao et al.. proposed that PDVs interacts with target cells through three mechanisms: endocytosis, membrane fusion, and binding to membrane receptors, based on non-plant-derived vesicles (NPDVs). However, the latter two are not currently observed in PDVs [10]. Numerous studies have shown that targeted cells internalized PDVs by macropinocytosis and endocytosis [43, 44]. In studies involving obesity intervention using turmeric-derived vesicles (TM-DVs), the endocytosis pathway was inhibited using chlorpromazine, genistein and EIPA, separately. The significant decrease in target cell uptake suggested the involvement of clathrin-mediated endocytosis, caveolae-mediated endocytosis, and macropinocytosis. Interestingly, when fluorescently labelled vesicles were used, the fluorescent signal detected on the cell membrane was much lower than in the cytoplasm, indicating endocytosis-driven internalization rather than membrane fusion [45]. Another study used the same technique to investigate the mechanism of PDVs internalization by target cells and found that *Lycium ruthenicum*-derived vesicles (LR-DVs) were taken up by PC12 cells via caveolae-mediated endocytosis and macropinocytosis, and the rate of uptake was concentration- and time-dependent [46].

It has to be admitted that compared to NPDVs, the study of the internalization mechanism of PDVs is still in its infancy and there are many questions that remain unanswered [47, 48]. For instance, what is the potential link between endocytosis and plant species or developmental stages? How do different molecules affect endocytosis through a complex regulatory network?

### Removal

So how should PDVs internalized into target cells be removed? Co-localization analysis showed that only a small fraction of PDVs enters the lysosome after cell internalization, implying a lysosomal escape ability to improve bioavailability [45]. Another study showed that grapefruit-derived vesicles (GF-DVs) co-localized with endosomes/lysosomes after uptake into HaCaT cells [49]. Therefore, we hypothesize that when target cells ingest PDVs, part of them will release its functional contents and exert their biological effects. And the other part of them will be degraded via the endosomal-lysosomal pathway.

## Pre-processing, isolation and characterization

Despite the many differences in PDVs compared to mammalian cell-derived vesicles, there are still many similarities as far as processing methods are concerned [50]. In this section, we describe recent advances in methods for the pre-processing, isolation, and characterization of PDVs **(**Table 1**)**.

**Table 1:** Nomenclature, pre-processing, separation and characterization of PDVs in recently reported (2020–2024)

Nomenclature (Based on MISEV 2023)	Nomenclature (Based on original reference)	Plant type	Pre-processing methods	Isolation methods	Characterization methods	References
PDEV	Plant extracellular vesicles	Sorghum	Tissue infiltration centrifugation	DGC^a^	TEM^i^, Cryo-EM^j^, SEM^k^, NTA^l^, DLS^m^	[51]
	Plant-derived extracellular vesicles	*Arabidopsis thaliana*	Tissue infiltration centrifugation	PBP^b^	TEM, NTA, DLS	[52]
	Plant-derived nano-and microvesicles	Salvia	Conditioned media	dUC^c^, SEC^d^	TEM, SEM, NTA	[53]
	Exosome-like extracellular vesicles	*Arabidopsis thaliana*	Tissue infiltration centrifugation	PBP, UF^e^, dUC	SEM, NTA	[54]
	Plant extracellular vesicles	*Arabidopsis thaliana*	Tissue infiltration centrifugation	dUC, IAC^f^	TEM, NTA	[55]
PDACDV	Exosome-like nanovesicles	Garlic	Tissue distruption	dUC, DGC	TEM, LDS^n^	[56]
	Plant-derived exosome-like nanovesicles (P-ELNs)	*Momordica charantia*	Tissue distruption	dUC	Bio-EM^o^, NTA	[56]
	Plant-derived exosome-like nanoparticles,	Turmeric	Tissue distruption	dUC	TEM, DLS	[45]
	Exosome-like nanoparticles	Grape	Tissue distruption	dUC	SEM, NTA	[57]
	Plant-derived extracellular vesicles	*Momordica charantia*	Tissue distruption	EP^g^	TEM, NTA	[58]
	EVs isolated from plants	*Citrus reticulata*	Tissue distruption	TFF^h^	SEM, DLS	[59]

^a^DGC: Density gradient centrifugation; ^b^PBP: PEG-based precipitation; ^c^dUC: Differential ultracentrifugation; ^d^SEC: Size exclusion chromatography; ^e^UF: Ultrafiltration; ^f^IAC: Immunoaffinity capture; ^g^EP: Electrophoresis; ^h^TFF: Tangential flow filtration; ^i^TEM: transmission electron microscopy; ^j^Cryo-EM: cryo-electron microscopy; ^k^SEM: scanning electron microscopy; ^l^NTA: nanoparticle tracking analysis; ^m^DLS: dynamic light scattering; ^n^LDS: laser diffraction spectrometry; ^o^Bio-EM: biological electron microscopy

### Pre-processing

**Fig. 4:**
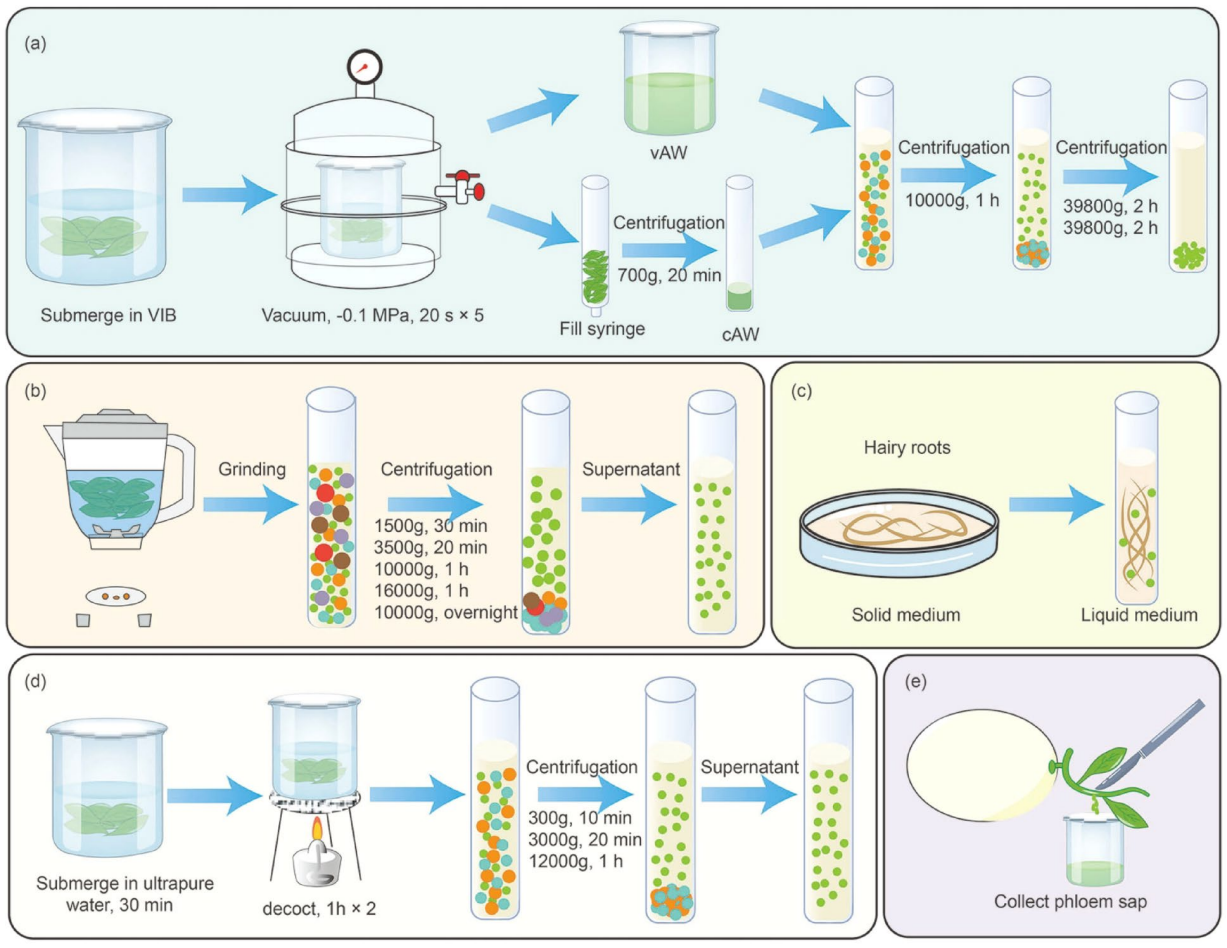
Pre-processing methods for PDVs. (**A**) Tissue-infiltration centrifugation; (**B**) Tissue-disruption; (**C**) Conditioned media method; (**D**) Decoction method; (**E**) Stem incision method. VIB, vesicle isolation buffer; vAW, vacuum-based apoplastic wash; cAW, centrifugation-based apoplastic was

The most common methods of plant tissue pre-processing used by researchers currently include tissue-infiltration centrifugation and tissue-disruption. Additionally, some special pre-processing methods have also been used **(**Fig. 4**)**.

#### Tissue-infiltration centrifugation

In plant science, tissue-infiltration centrifugation is often widely used as a starting technique for obtaining plant material, by which apoplastic fluids are obtained for variable omics analyses, including metabolomics [60, 61]. The apoplast is the space located between the plant cell’s plasma membrane and its outer surface. The apoplast contains cell wall polysaccharides, proteins, polyphenols, and ions [62]. Isolation of PDVs from apoplastic fluids was first accomplished in sunflower [63]. Following this, Rutter et al.. introduced a thorough procedure for isolating EVs from the apoplastic washes of *Arabidopsis thaliana* leaves, setting a standard for PDVs pre-processing [24, 64]. On this basis, Adekanye et al.. re-optimized the tissue-infiltration centrifugation method and used it in sorghum and *Arabidopsis thaliana* [51]. They first submerged the plants in vesicle isolation buffer. Part of the tissues were vacuum infiltrated to obtain vacuum apoplastic wash (vAW), and the other part of the tissues were centrifuged to collect centrifuge apoplastic wash (cAW). Afterwards, vAW and cAW were mixed and centrifuged at 10,000 × g for 1 h to obtain PDVs pelleting [65]. Compared to tissue-disruption, PDVs obtained after tissue-infiltration centrifugation is of higher purity and is defined closer to vesicles than to particles. Furthermore, by maintaining tissue integrity as much as possible, the need for downstream purification steps is minimized [66]. No studies have focused on the effect of specific buffer composition. Whether pH, metal ions, etc. affect the stability and size of PDVs still needs to be further explored.

#### Tissue-disruption

Tissue-disruption is a method that uses mechanical force to disrupt plant tissues to obtain plant juice and thus extract PDVs, and is currently the most widely used pretreatment method [56, 67–69]. We reviewed and summarized the four steps of the tissue-disruption method: (1) extraction of plant juice using a blender, (2) removal of large particles and cellular debris using multi-step low-speed centrifugation, (3) removal of the supernatant obtained using high-speed centrifugation to obtain the pellet, and (4) resuspension of the pellet in phosphate-buffered saline (PBS). It is clear that tissue-disruption yields more PDVs compared to tissue-infiltration centrifugation, which is why most studies use tissue-disruption for pre-processing [70]. During subsequent purification, vesicles formed by spontaneous membrane resealing after disruption of cellular and organelle membranes are difficult to distinguish from normal PDVs. And the vesicle size is larger than that of vesicles from tissue-infiltration centrifugation method [71]. Tissue-disruption may also trigger plant defense mechanisms that alter vesicle composition [72]. Moreover, Garaeva et al.. found that spherical individual particles corresponding to the vesicle topology with diameters between 50 and 120 nm and heights between 30 and 60 nm, as well as some small particles with heights of about 15 nm, were observed by atomic force microscopy (AFM) when using the tissue-disruption. In contrast, cryo-electron microscopy (cryo-EM) observed an average size of 41 ± 13 nm for circular vesicles, suggesting that the tissue-disruption method generates a large number of non-vesicle particles [73].

#### Other pre-processing methods

There are also special pre-processing methods that deserve the attention of researchers. The decoction method uses water as a solvent and extracts the components in a boiling state [74]. Li et al.. first soaked American ginseng for 30 min, after which the decoction was boiled for 1 h. The filtrate was concentrated and subsequently isolated using differential ultracentrifugation (dUC) [75]. The PDVs obtained by this method had a stronger therapeutic effect than those obtained by the conventional decoction method [76]. It has also been suggested that in studies of melon-derived vesicles (ML-DVs), the phloem sap was collected using the stem incision method and subsequently isolated using size exclusion chromatography (SEC) [77]. Vestuto et al.. collected hairy roots of Salvia, first cultured on solid medium and later transferred to liquid medium, and used dUC and SEC to isolate PDVs from the liquid medium [53].

### Isolation

**Fig. 5:**
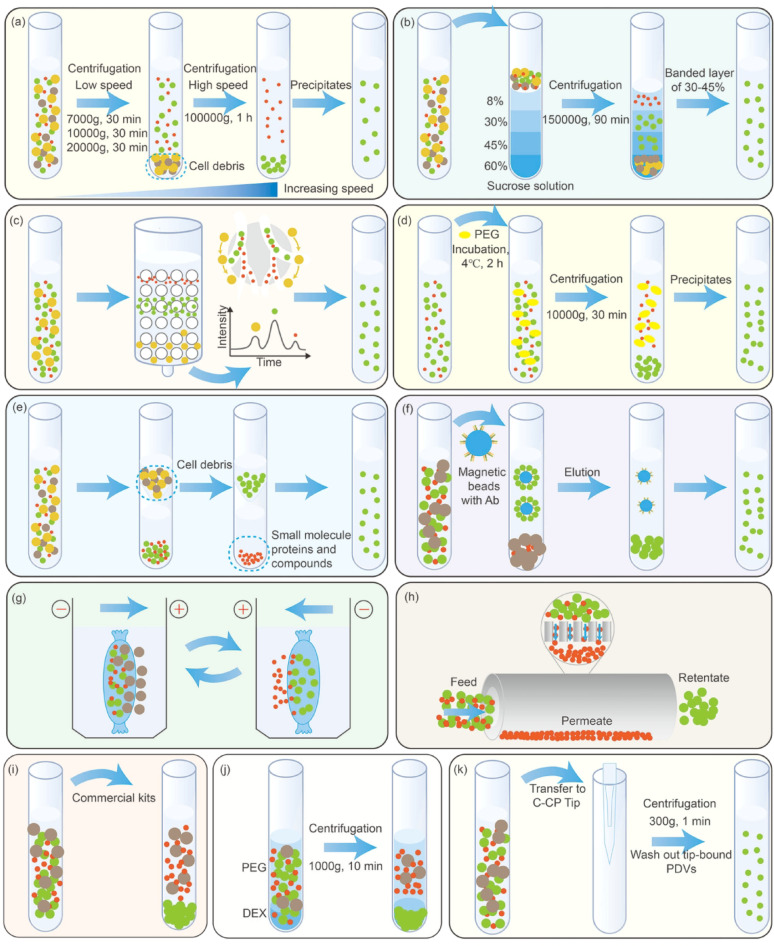
Isolation methods for PDVs. (**A**) differential ultracentrifugation; (**B**) density gradient centrifugation; (**C**) size exclusion chromatography; (**D**) PEG-based precipitation; (**E**) ultrafiltration; (**F**) immuno-affinity capture; (**G**) electrophoresis; (**H**) tangential flow filtration; (**I**) kits; (**J**) aqueous two-phase isolation method; (**K**) C-CP fibre spin-down tip approach. PEG, polyethylene glycol; DEX, Dextran; C-CP, capillary-channeled polymer.

Considering the functionality and potential clinical applications of PDVs, obtaining high quality and yield of PDVs remains a challenge. Therefore, we describe the isolation techniques that are often utilized by researchers **(**Fig. 5**)**.

#### dUC

As same as the isolation of vesicles of eukaryotic origin, dUC is the most commonly used technique for the isolation of PDVs due to the reason that it seems to offer the greatest advantages (i.e., simplicity, economy) [78, 79]. Shkryl et al.. demonstrated the classical steps for PDVs isolation by dUC: They used a series of low-speed centrifugation steps (7,000 × g, 10,000 × g, and 20,000 × g for 30 min each) to remove cellular debris, followed by high-speed centrifugation (100,000 × g for 1 h) to allow for the precipitation of the PDVs [57]. Inevitably, during centrifugation, PDVs may precipitate with protein aggregates. Moreover, this is a time-consuming, operator- and equipment-sensitive process. The acceleration, rotor type and intrinsic characteristics of the centrifuge are determining factors that affect the ultimate efficiency of EVs isolation [80].

#### Density gradient centrifugation (DGC)

DGC introduces an inert centrifugal medium into the centrifugation system so that the PDVs is transferred to the appropriate density gradient interval by ultracentrifugation [81]. The medium for density gradient centrifugation is mainly sucrose and iodixanol [82]. As an example of using sucrose as a medium, a gradient of sucrose (8%, 30%, 45%, 60%) was used, followed by centrifugation at 150,000 × g for 90 min. After that, about 30–45% of the band layer was flushed with PBS, centrifuged again, and a precipitate was obtained [83]. The pre-processing methods also affect vesicle density, e.g., vesicles obtained by tissue-infiltration centrifugation and tissue-disruption are distributed in different sucrose density layers [71]. Some researchers have also recommended using iodixanol rather than sucrose because it is isotonic at all concentrations [84]. Despite the improvement in purity and separation efficiency compared to dUC, DGC still has some disadvantages, such as relatively complicated operation and expensive equipment [85].

#### SEC

SEC uses a porous polymer matrix to collect several EVs fractions [86]. SEC has several distinct advantages over dUC. Firstly, SEC relies on gravity, whereas by dUC the separated vesicles may be subjected to high shear stresses that can destroy surface molecules. Next, SEC avoids another disadvantage of dUC, which is the aggregation and morphological changes of vesicles [87]. Of course, SEC has some disadvantages, such as low recovery, difficulty in reusing columns, small amount of samples that can be processed at one time, and inability to avoid the interference of impurities with the same size as PDVs [86, 88, 89]. Vestuto et al.. used dUC and SEC to isolate PDVs from salvia hairy root, respectively, and they found that both methods maintained the integrity of the PDVs protein cargo with comparable vesicle sizes. However, SEC obtained much lower yields than dUC [53].

#### PEG-based precipitation (PBP)

Polyethylene glycol (PEG) can precipitate EVs by inducing dehydration and increasing their solute concentration [90]. In the latest study, Jokhio et al.. used PEG 8000 and NaCl to isolate *Arabidopsis thaliana*-derived vesicles (AT-DVs) from apoplastic washing fluid (AWF). Specifically, they mixed AWF with PEG 8000, added NaCl to obtain 0.5 M salinity, incubated at 4 °C for 2 h and centrifuged at 10,000 × g force for 30 min at 4 °C. PEG-EVs agglomerates were re-suspended using PBS, after which they could be characterized [52]. The advantage of PEG-based precipitation over ultracentrifugation is that it is easy to use and greatly reduces isolation-related production costs. However, there are still some challenges, such as low specificity [91]. Optimization of the extraction process showed that the best yield and activity of PDVs was achieved at 10% PEG concentration, pH 5, and centrifugation temperature of 4℃ [92].

#### Ultrafiltration (UF)

UF can be defined as filtration under pressure through filters with minute pores, thus allowing the separation of large molecules from smaller ones [93]. In a study comparing UF and PBP isolation of PDVs, PDVs isolated by UF maintained the best morphology. However, it had the most protein contamination compared with other methods [54]. Therefore, a number of studies have used UF in combination with other isolation methods, such as dUC and Sects. [94, 95].

#### Immuno-affinity capture (IAC)

IAC is considered an advanced method for purifying specific classes of PDVs [96]. Typically, antibodies against PDVs surface proteins are covalently linked to magnetic beads through biotinylation [97]. Since the amino terminus and carboxyl terminus of the TET8 protein are inside the vesicle [98], He et al.. designed a native antibody that specifically recognized the large exposed extravesicular loop, EC2 domain, of TET8. By means of this, TET8-positive PDVs were successfully isolated by IAC [99]. Subsequently, Wang et al.. used the same antibody as He et al.. for direct coupling to agarose beads and isolation of PDVs by IAC [55]. PEN1 antibody was also used for the detection of sorghum-derived vesicles (SG-DVs) [51]. Interestingly, one study purified PDVs by immobilizing Concanavalin A (a widely used lectin) on magnetic beads so that bacteria could be removed by negative enrichment [100]. Additionally, Exo70E2 proteins or its paralogues (e.g. Exo70A1) may also be used for IAC, but further studies are needed to confirm this [101].

#### Electrophoresis (EP)

EP refers to the isolation of charged molecules based on their mobility in an electric field [102]. Only a few studies have used electrophoresis to isolate vesicles [103]. Yang et al.. developed a technique that combines electrophoresis and dialysis to isolate lemon-derived vesicles (LM-DVs) that are similar in size, shape and number to those isolated by ultracentrifugation. EP is much more time-efficient and does not require bulky or complicated equipment [104]. Subsequently, the same method was used for the isolation of *Momordica charantia*-derived vesicles (MC-DVs) [58]. In addition, capillary electrophoresis can also be used for development and process control of PDVs isolation [101].

#### Tangential flow filtration (TFF)

Conventional UF can lead to cake formation and sample aggregation that can block the membrane. In contrast to UF, TFF has the significant advantage of allowing waste to flow in a direction orthogonal to the sample flow, thus preventing membrane blockages [105]. In the isolation of PDVs, TFF is often used in conjunction with other isolation methods, e.g., isolation of *Citrus reticulata*-derived vesicles using dUC, UF, and TFF [59]. The same combination of isolation methods can also be used for the isolation of *Aster yomena*s-derived vesicles (AY-DVs) and *Aloe vera*-derived vesicles (AV-DVs) [106, 107].

#### Kits

Commercial kits based on polymer precipitation have now been used in the isolation of PDVs, Sharma et al.. removed cellular debris by centrifugation and added ExoQuick-TC reagent to precipitate PDVs [108]. Nevertheless, ISEV does not encourage the use of kits, which may precipitate many free proteins. Furthermore, compared to dUC, the amount of total mRNA obtained by the kits is less, which is not favorable for subsequent analyses [8, 109].

In addition, aqueous two-phase isolation method and capillary-channeled polymer (C-CP) fibre spin-down tip approach also be used for the isolation of PDVs [110, 111]. Improving the quality and yield of PDVs will not only advance the research in related fields, but also provide new solutions for industrial applications.

### Characterization

#### Particle number concentration

For the assessment of PDVs particle number concentrations, researchers often use dynamic light scattering (DLS) and nanoparticle tracking analysis (NTA) [51]. As an example, one study used NTA to determine that the concentration of particles in *Aloe vera* was 6.5 ± 5.7 × 10^8^ particles/gram of raw material [112]. The metric is commonly reported and utilized for standardizing assay inputs, measuring assay outputs and in vivo dosing, but it frequently lacks reliability because of its non-specificity [8].

#### Size

For particle size measurements, DLS, NTA, microfluidic resistive pulse sensing (MRPS) and cryo-EM can be used [73, 113]. It is becoming increasingly clear that many particle sizes show asymmetric left-skewed distributions, e.g. *Arabidopsis thaliana*, olive vegetation [71, 114]. Research conducted previously has demonstrated that particle size is linked to various factors beyond just plant type, such as whether tissue-infiltration centrifugation or tissue-disruption is used, the plant organ, the pH of the supernatant at the time of isolation, and whether it was derived from plant calluses or plant berry juice, etc [57, 71, 115–117]. For instance, the mean diameter of potato root-derived vesicles (164.6 ± 7.3 nm) was significantly larger than that of potato peel-derived vesicles (132.2 ± 2.0 nm) [117]. Notably, certain studies have used PBS and 10% fetal bovine serum (FBS) to mimic in vitro and in vivo environments. They determined vesicle stability by detecting changes in particle size distribution and zeta potential [118].

#### Morphology

Various microscopy techniques, such as TEM, scanning electron microscopy (SEM), Cryo-EM, scanning probe microscopy (SPM), biological electron microscopy (Bio-EM) and AFM were used for the morphological characterization of PDVs [68, 70]. When the morphology was also observed using TEM (the most commonly used EM technique in the PDVs), the PDVs generally appeared spherical, with a very small portion appearing saucer-shaped or cup-shaped. In contrast, when Cryo-EM was used, it generally appeared spherical [25, 56, 114, 119, 120]. Morris et al.. concluded that both TEM and SEM require a vacuum environment, which leads to vesicle shrinkage and morphological changes, and to mitigate this effect, Cryo-EM is recommended. Also, it is capable of distinguishing between single, double and multi-layer vesicles [114, 121].

#### Proteins

Techniques for analyzing proteins mainly include specific protein detection, protein quantification and proteomics analysis. To identify the specific proteins in PDVs, Western blotting is commonly used. Several proteins, such as TET8, PEN1, BiP, PDR8 and HSP70 have been used as protein markers for PDVs [116]. Recent studies have shown that Western blotting of AT-DVs using a mammalian exosomal kit revealed surface marker tetrapan proteins (CD9, CD63 and CD81), as well as endosomal sorting complex required for transport (ESCRT)-associated proteins (TSG101 and ALIX). This suggests that PDVs and mammalian-derived vesicles share a partially identical molecular composition [52]. The quantitative analysis of proteins mainly includes the bicinchoninic acid (BCA) method and Qubit Fluorometric Quantitation, with the BCA assay being the most commonly used [71, 112, 116]. It is worth mentioning that the total amount of proteins in PDVs obtained by tissue destruction was higher than in tissue-infiltration centrifugation [71]. A variety of PDVs have been analyzed for proteomics, such as *Arabidopsis thaliana*, *Catharanthus roseus*, spruce, garlic, pomegranate and tangerine [24, 122–126]. The proteomics technique commonly used is liquid chromatography-mass spectrometry (LC-MS), which facilitates the recognition of a large number of proteins in intricate EV samples [127]. For mass spectrometry, the PDVs should be purified at least by density gradient [51].

The most studied PDVs proteins are TET8 and PEN1, both of which are plasma membrane proteins [128]. TET8 belongs to the tetraspanin family and shares structural similarities with the mammalian exosome marker CD63, which has been identified in AT-DVs [52, 129]. PEN1, also known as SYP121, was originally found to be associated with the control of ion channels in the plasma membrane of stomatal guard cells [130]. Following the collection of 40,000 g and 100,000 g of products (referred to as P40 and P100 fractions) from *Arabidopsis thaliana* apoplast. It was observed that TET8-positive PDVs were enriched in the P100 fraction, while PEN1-positive PDVs were enriched in the P40 fraction, indicating that the two types of EVs may be different subclasses of PDVs [24, 99]. Also, after labelling PDVs using fluorescence-tagged fusion proteins (TET8-GFP and mCherry-PEN1), they did not co-localize, further confirming this speculation [99]. Further studies revealed that the *Arabidopsis thaliana* MVBs marker Rab5-like guanosine triphosphatase (GTPase) ARA6 partially co-localized with TET8, suggesting that TET8-positive PDVs may originate from MVBs. However, PEN1 did not co-localize with ARA6, suggesting that PEN1 and TET8-positive PDVs have different biogenesis pathways [99, 129]. So, how does TET8 affect PDVs biogenesis? Liu et al.. showed that EV sphingolipids consist almost purely of glycosylinositolphosphoceramides (GIPCs), and that *Arabidopsis thaliana* TET8 knockout mutant cells had a lower number of GIPCs and secreted fewer PDVs [54]. Subsequently, they identified TET8 as a sphingolipid carrier, thereby regulating the export of GIPCs from the Golgi apparatus. And they elucidated the molecular mechanisms involved in GIPC transport and PDVs biogenesis [98]. As a protein associated with the EXPO pathway, Exo70E2 and its paralogues have attracted much attention [31, 131]. There is increasing evidence that PEN1 appears to be closely related to the EXPO pathway, e.g. Exo70A1 has a significant interaction with PEN1 [132]; Exo70B2 and PEN1 proteins have the ability to directly interact with each other [133]. However, there is no direct evidence that PEN1 is involved in the EXPO pathway.

Another reason TET8 and PEN1 have received so much attention is that researchers want to develop general protein markers for PDVs [134]. There is no doubt that certain PDVs proteomes are partially conserved, providing a theoretical basis for development in this direction. Nine common proteins, such as ATP synthase A, HSP70 and an ABC transporter G family member, were found in the proteome of salvia-derived vesicles and AT-DVs. These proteins, which are common in phylogenetically distant species, may represent a conserved component of the PDVs proteome in plants [53]. Conservation of SG-DVs and AT-DVs proteomes was also found, such as the shared proteins PEN1, PATL1, and TET8 [51]. Using immune-affinity capture, they found that PEN1 may be a useful protein marker for identifying SG-DVs and AT-DVs [51]. However, the heterogeneity of the proteome poses an obstacle to the development of general protein markers and further studies are still needed [116].

#### Lipids

The lipid bilayer of PDVs is mainly composed of sphingomyelin, cholesterol and ceramides, which perform important roles in membrane formation, cell signaling and vesicle release [50]. Therefore, it becomes particularly significant to study the amount and specific composition of lipids from different plant species sources and from different subgroups of PDVs. In terms of simple quantitative analysis, the total lipid assay kit is one of the common choices used by researchers, and Ramírez et al.. used this method for AV-DVs [112]. Indeed, as far as the analysis of lipidomics is concerned, LC-MS is the most commonly used method, which is consistent with the analysis of NPDVs [114, 135, 136].

In lipidomic analysis of PDVs, the phosphatidylethanolamine (PE)/PA ratio has received much attention. In Rutaceae, orange-derived vesicles (OG-DVs) contained PE (40%), phosphatidylcholine (PC; 25%), phosphatidylinositol (PI; 12%), and PA (5%). Similarly, GF-DVs contained PE (45.52%), PC (28.53%) and PA (2.50%) [43, 137]. TM-DVs have been found to also possess high PE/PA, according to recent studies [135]. In contrast, olive vegetation and tomato reversed this ratio, showing high PA [22, 114]. Notably, some PDVs also contain unique lipid components. Ou et al.. found that *Catharanthus roseus*-derived vesicles (CR-DVs) contain more than 30% ether-phospholipids [122], which is crucial for maintaining the balance of mitochondrial reactive oxygen species (ROS) and regulating ferroptosis [138, 139].

Researchers have shifted their focus from just analyzing lipid composition to exploring their biological roles. The novel finding by Chen et al.. demonstrated that lipids, but not proteins or RNAs, in GG-DVs are the active biomolecules that inhibit the activity of NLRP3 inflammasome activation [140]. Garlic chive-derived vesicles (GC-DVs) contain the phospholipid 1,2-dilinoleoyl-sn-glycero-3-phosphocholine, which has been identified as an inhibitor of NLRP3 [124]. In addition to anti-inflammatory effects, PA from GG-DVs induces Foxa2 expression and prevents Foxa2 phosphorylation to prevent high-fat diet-induced insulin resistance [141].

#### Nucleic acids

Studies have shown that PDVs are enriched with nucleic acids, but mainly RNA [134]. Baldrich et al.. studied small RNAs (sRNAs) in AT-DVs, which mainly consist of microRNAs (miRNAs) and small interfering RNAs (siRNAs). They also identified a previously overlooked class of ‘tiny RNAs’ (10 to 17 nt) highly enriched in PDVs, which may be degradation products of mRNAs, primary miRNAs, siRNAs, tasiRNAs, and hcRNAs [142]. Sequencing of vesicular sRNAs from broccoli, pomegranate, apple, and orange showed all contained four common miRNA families (miR-159, miR-162, miR-166, and miR-396), suggesting similarities in PDV-loaded endogenous miRNAs [116].

Diversity of PDV-associated RNAs points to a wide range of biological functions [41]. It was found that miRNAs loaded in GS-DVs induced neural differentiation of bone marrow mesenchymal stem cells (BMSCs) [143]. MiRNA164a/b-5p in tomato-derived vesicles (TMT-DVs) modulates the Keap1/Nrf2 signaling pathway and alleviates restenosis after vascular injury [144]. Furthermore, Wang et al.. discovered that Selenium enrichment increased miR-167a expression in vesicles from broccoli and triggered apoptosis in human pancreatic cancer cells [145].

#### Metabolites

Detection of metabolites is generally carried out by LC-MS and nuclear magnetic resonance (NMR). The commonly used LCs include high-performance liquid chromatography (HPLC), ultra-performance liquid chromatography (UPLC), and other methods [11, 43, 107]. Previous studies have shown that certain specific metabolites will be enriched in PDVs to perform specific physiological functions [126, 146]. Shkryl et al.. found that grape-derived vesicles were enriched in δ-viniferin, possibly due to their strong hydrophobicity [57]. Neral, detected in *Solanum nigrum*-derived vesicles, is a typical monoterpene found in lemons with anti-inflammatory properties [147]. Balloon flower -derived vesicles contain high levels of platycodin D, which is known to regulate inflammation and ameliorate systemic toxicity [148]. In addition to exerting anti-inflammatory effects, certain metabolites can also exert anti-tumor effects, such as ginsenosides in GS-DVs [11]. Notably, Kocholatá et al.. detected the presence of secondary metabolites (i.e., nicotine and anabasine) from tobacco calli and apoplastic fluid-derived vesicles, whereas cell culture-derived vesicles were not detected [149].

## PDVs and periodontitis treatment

**Fig. 6:**
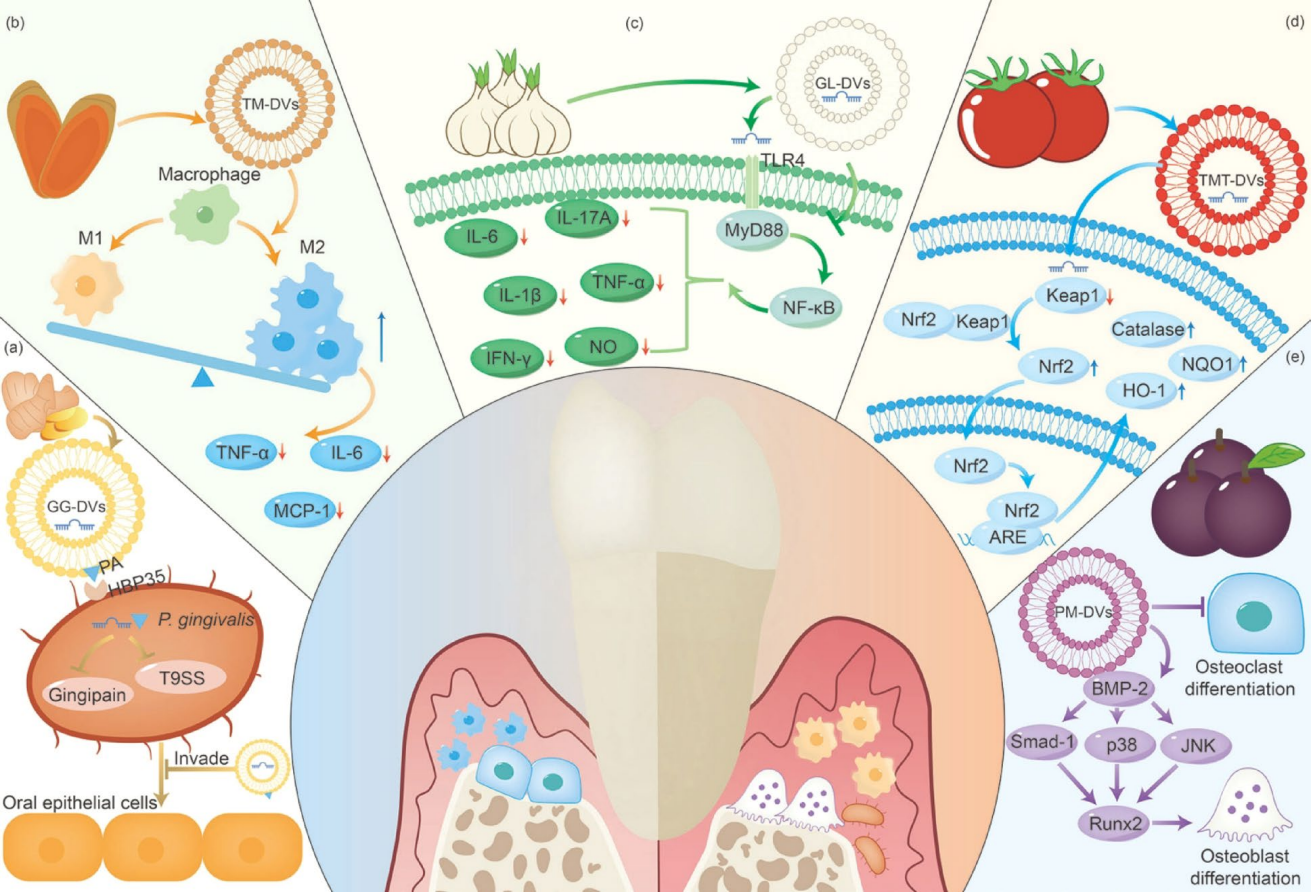
The potential mechanism of PDVs for periodontitis treatment

The oral microbial ecosystem is the habitat of a wide variety of bacteria and viruses, although little is known about the key factors that maintain a stable balance [150, 151]. However, when dysbiosis occurs, the host fights against the dysbiosis through a variety of weapons such as epithelial cells, dendritic cells, natural killer cells, T cells and B cells, which lead to localized immune dysregulation in periodontal tissues through a number of pathways. This results in an excessive inflammatory response that creates a microenvironment favorable to pathogenic bacteria [151]. Due to the unique advantages of PDVs, they have shown great potential for application in maintaining the dynamic balance of oral microecology, remodeling the immune microenvironment, regulating inflammatory response, regulating oxidative stress and periodontal tissue regeneration **(**Fig. 6; Table 2**)**.

**Table 2:** Possible PDVs for periodontitis treatment

Physiological function	Plant type	Target	Potential mechanisms	References
Maintain the dynamic balance of oral microecology	Ginger	Porphyromonas gingivalis (Pg)	PA directly interacts with HBP35 protein in Pg, leading to inhibition of Pg growth	[14]
	Tomato	Fusobacterium nucleatum and Lactobacillus spp.	Inhibit growth of Fusobacterium nucleatum and promote the growth in Lactobacillus spp.	[152]
	Garlic	Staphylococcus aureus	Kill the bacteria and modulate the immune response	[56]
	Dandelion	Staphylococcus aureus	Bind stably and directly to the exotoxin	[153]
	Ginger	Lactobacillus rhamnosus (LGG)	Promote growth	[154]
	Tartary buckwheat	LGG and Escherichia coli	Promote growth	[155]
	*Portulaca oleracea*	Lactobacillus reuteri	Enhance growth and elevate indole derivative levels	[156]
	Ginger	Lactobacilli spp.	Augment the abundance of Lactobacilli spp.	[15]
	Mulberry bark	Listeria monocytogenes	Inhibit growth	[157]
	Garlic	Helicobacter pylori (Hp)	Downregulated the abundance of Hp	[158]
Remodel the immune microenvironment	Ginseng	Macrophage	Inhibit M1 macrophage polarization and promotes M2 macrophage polarization	[159]
	Garlic	Macrophage	Inhibit M1 macrophage polarization and promote M2 polarization	[160]
	Goldenberry	Macrophage	Inhibit M1 macrophage polarization and promote M2 polarization	[161]
	Ginger	Macrophage	Promote macrophage M2 polarization by downregulating NF-κB expression	[162]
	*Rehmanniae radix*	Macrophage	Promote M2 macrophage polarization	[163]
	*Pueraria lobata*	Macrophage	Promote M2 macrophage polarization	[164]
	Turmeric	Macrophage	Promote M2 macrophage polarization	[165]
	Ginger	Macrophage	Promote M2 macrophage polarization by modulating the PI3K-AKT pathway	[166]
	*Aster yomena*	Dendritic cells (DCs)	Inhibit the expression of surface molecules, increase the internalization of extracellular antigens, and suppress the antigen-presenting ability	[107]
	Broccoli	DCs	Enhance AMPK signaling and promote induction of AMPK-activated anti-inflammatory factors	[167]
	*Portulaca oleracea*	CD4 + T cells	Lead to the reprogramming of conventional CD4 + T cells into double-positive CD4 + CD8 + T cells	[156]
	Garlic	γδ T cells	Induce the endogenous expansion and activation of γδ T cells	[168]
Regulate inflammatory response	Ginger	NLRP3 Inflammasome	Inhibit NLRP3 inflammasome-mediated IL-1β and IL-18 secretion and pyroptosis	[140]
	Ginger	Intestinal epithelial cell	Reduce the pro-inflammatory cytokines (TNF-α, IL-6 and IL-1β), and increase the anti-inflammatory cytokines (IL-10 and IL-22)	[169]
	Ginger	Caco-2 Cells	Inhibit the expression of NF-κB and an array of inflammatory cytokines expression	[170]
	Ginger	SARS-COV-2	Inhibit Nsp12 and spike gene expression	[171]
	Garlic	NLRP3 Inflammasome	Downregulate NLRP3 protein levels	[160]
	Garlic	Caco-2 cells	Regulate the TLR4/MyD88/NF-κB signaling pathway and the secretion of inflammatory cytokines	[158]
	Garlic	THP-1 macrophages	Inhibit TNFα, IL-6, and IL-1β production	[172]
	Apple	Macrophage	Negatively regulate the production of pro-inflammatory cytokines by inhibiting NF-kb pathway	[173]
	Apple	Human primary dermal fibroblasts	Negatively regulate Toll-like receptor 4 activity, which downregulates the NF-κB pro-inflammatory pathway	[174]
	Lemon	THP-1 M0 macrophages	Reduce the expression of IL-6 and TNF-α, while increase the levels of IL-10	[175]
	Lemon	LPS-induced macrophages	Inhibit the ERK1/2-NF-κB signaling pathway	[176]
	*Momordica charantia*	NLRP3 Inflammasome	Downregulate the gene expression of NLRP3 and protein expression of NLRP3 and pro-IL-1β	[58]
	Shiitake mushroom	NLRP3 Inflammasome	Inhibit NLRP3 inflammasome activation and suppress the secretion of IL-6	[177]
	Garlic chive	NLRP3 Inflammasome	Inhibit NLRP3 inflammasome activation	[124]
	Grapefruit	Macrophage	Induce HO-1 and IL-10 expression, which in turn inhibited the secretion of the inflammatory cytokines IL-1β and TNF-α	[137]
	Cabbage	RAW264.7 cells	Inhibit IL-6, IL-1β production	[94]
	*Solanum nigrum*	RAW264.7 cells	Inhibit IL-6 production	[147]
	American ginseng	Macrophage and RAW264.7 cells	Reduce macrophage migration and regulate inflammatory factor (NO, TNF-α, IL-6, IL-10) secretion in RAW 264.7 cells	[75]
	*Citri reticulate pericranium*	RAW264.7 cells	Inhibit the level of inflammatory markers like NO and inflammatory biological factors (IL-6, IL-1β and TNF-α)	[178]
	Turmeric	Mice colitis models	Regulate the expression of the pro-inflammatory cytokines	[179]
	Taraxacum officinale	Rat model of intermittent hypoxia-induced hypertension	Inhibit the production of IL-1β, IL-6 and TNF-α.	[180]
	*Aloe vera*	RAW264.7 and THP-1 M0 macrophages	Decrease the secretion of pro-inflammatory cytokines TNFα, IL-1β, and IL-6	[112]
	Hemp	DSS-induced mice colitis	Attenuate NF-κB activation and oxidative stress markers	[181]
	*Arbutus unedo*	RAW264.7 cells	Selectively inhibit JAK/STAT1-COX-2 signaling and decrease the secretion of IL-1β and IL-6	[182]
Regulate oxidative stress	Ginger	Chondrocytes	Attenuate oxidative stress in TBHP-induced chondrocytes by activating the Nrf2 pathway	[183]
	*Aloe vera*	HaCaT cells	Activate the Nrf2 pathway	[106]
	Ginger	Hepatocytes	Reduction of ROS production by activating Nrf2 nuclear translocation via the TLR4/TRIF pathway	[184]
	Blueberry	Rotenone-treated HepG2 cells	Accelerate the translocation of Nrf2 from the cytoplasm to the nucleus and reduce ROS production	[185]
	*Lycium ruthenicum*	Aβ-induced oxidative stress in HT22 cells	Promote the nuclear translocation of Nrf2 and upregulate the expression of HO-1 and NQO1	[186]
	Tomato	Vascular smooth muscle cells	Upregulate Nrf2 expression in the nucleus	[144]
	Carrot	H9C2 Heart-Derived Cardiomyoblasts	Suppress apoptosis caused by oxidative stress by inhibiting the decrease in antioxidative proteins	[187]
	*Momordica charantia*	Ulcerative colitis in C57BL/6 Mice	Upregulate SOD, GSH, GSH-Px, and CAT levels, thereby inhibiting lipid oxidation and scavenging hydrogen peroxide	[188]
	Grape and tomato	H2O2 stimulated L-02 cell model	Increase SOD, CAT and GSH-PX activities and decrease ROS production	[189]
	*Robinia pseudoacacia*	Mice gastric mucosa	Downregulate the expression of NOX4 and ALOX5, which drive ROS production and lipid peroxidation, respectively	[190]
	*Citri reticulate pericranium*	RAW264.7 cells	Increase the levels of GSH and promote the activity of antioxidant markers such as SOD, CAT, and GR	[178]
	Hemp sprout	Non-alcoholic fatty liver disease mouse models	Decrease the oxidative stress markers, such as 3-NT and TLR4	[191]
	Pomegranate	Small intestine in alcohol-induced mice	Reduce the iNOS, CYP2E1, and 3-NT protein expression	[192]
	Ginseng	HaCaT cells	Decrease the ROS levels induced by UV irradiation	[193]
	*Citrus limon*	Mesenchymal stromal cells	Reduce ROS production	[194]
	Tea	HepG-2 cells	Reduce ROS production	[195]
	Strawberry	Adipose-derived mesenchymal stem cells (ADMSCs)	Reduce ROS production	[196]
	Blueberry	EA.hy926 Cells	Reduce ROS production	[197]
	*Opuntia ficus*	SH-SY5Y cells	Reduce ROS production	[198]
	*Rehmanniae radix*	RAW264.7 cells	Reduce ROS production	[163]
Promote periodontal tissue regeneration	Grape	Lgr5hi intestinal stem cells	Induce proliferation of intestinal stem cells	[199]
	Ginseng	Bone marrow derived mesenchymal stem cells (BMSCs)	Induce neural differentiation and angiogenic	[143]
	*Aloe vera*	HaCaT and HDF cells	Promote the migration of HaCaT and HDF cells	[106]
	*Aloe saponaria*	HDF cells and HUVECs	Promote the migration of HDF cells and angiogenesis	[200]
	*Lithospermum erythrorhizon*	HDF cells	Promote HDF cells proliferation	[201]
	Grapefruit	HaCaT cells and HUVECs	Stimulate the migration of HaCaT cells and display favorable effects on angiogenesis capacities of the HUVECs	[202]
	Wheat	HDF, HUVECs, and HaCaT cells	Promote cell migration and angiogenesis	[203]
	*OPUNTIA ficus-indica*	HDF cells	Promote the migration of HDFs	[119]
	Ginseng	Osteoclast	Inhibit RANKL-induced osteoclast differentiation	[204]
	Plum	Osteoclast and osteoblast	Inhibit osteoclast differentiation and promote osteoblast differentiation through the BMP-2/MAPK/Smad-1-dependent Runx2 pathway	[205]
	Yam	Osteoblast	Promote proliferation, differentiation and mineralization through activation of the BMP-2/p-p38-dependent Runx2 pathway	[206]
	Apple	Osteoblast	Stimulate osteoblast differentiation through the BMP-2/Smad1 and BMP-2/MAPK signaling pathways	[207]
	*Cissus quadrangularis*	Human bone mesenchymal stem cells (hBMSCs)	Promote hBMSCs differentiation and mineralization both in vitro and in ovariectomized (OVX) induced osteoporotic rats	[208]
	*Rhizoma drynariae*	hBMSCs	Facilitate mRNA and protein expression of bone morphogenetic protein 2 and runt-related transcription factor 2 via the ERa signaling pathway	[209]

### Maintain the dynamic balance of oral microecology

In fact, oral microorganisms co-evolve with their hosts and are maintained through bidirectional interactions between the microbiome and the host to achieve harmonious coexistence, thus establishing a dynamic balance in oral microecology [210]. It is now widely recognized that periodontitis is not caused by one or a few pathogenic microorganisms, but rather is the result of the synergistic action of an abnormal microbial community, also known as the polymicrobial synergy and dysbiosis (PSD) model [211]. Therefore, from an allopathic point of view, the use of PDVs is necessary to re-establish the dynamic balance of oral microecology.

#### *P. gingivalis*

*P. gingivalis* is an anaerobic Gram-negative bacterium, which, as one of the most widely studied pathogens of periodontitis, disrupts the delicate balance between the various members of the oral microbial community [212]. Previous studies have shown that GG-DVs can reshape the gut microbiota and modulate host physiology [154]. Therefore, Sundaram et al.. explored the role of GG-DVs in inhibiting *P. gingivalis* [14]. PA in vesicles has been reported to inhibit *P. gingivalis* growth by directly interacting with the HBP35 protein in *P. gingivalis*. The miRNA and PA in the vesicles were also found to inhibit the virulence activity of Gingipain and the type IX secretion system. Furthermore, in vivo experiments demonstrated that GG-DVs inhibited *P. gingivalis*-induced bone loss [14]. However, this study did not examine the effect of GG-DVs on the established microecology and further studies are needed.

#### *Fusobacterium nucleatum* (*F. nucleatum*)

Similar to *P. gingivalis*, *F. nucleatum* is closely associated with the onset and progression of periodontitis [213, 214]. Recent studies have shown that TMT-DVs taken up by *F. nucleatum* exert their antimicrobial effects via lipid components. In addition, the vesicles also promote the growth of *Lactobacillus* spp. such as *L. acidophilus*, *L. plantarum*, *L. reuteri*, *B. bifidum*, *B. breve*, and *B. longum* [152]. In conclusion, this study highlights the potential application of TMT-DVs in balancing the gut microbiome, providing new insights into balancing oral microecology [152].

#### *Staphylococcus aureus* (*S. aureus*)

*S. aureus* is considered a pathogen with moderate evidence of triggering periodontitis [215]. Actually, PDVs was not used by researchers for *S. aureus* until recently; however, mainly for the treatment of wounds [56, 153]. Tan et al.. showed that dandelion-derived vesicles were able to neutralize *S. aureus* exotoxins. Also, the presence of plasma, NaCl, or FBS interfered minimally with the affinity, further demonstrating the strong affinity between the two [153]. Besides binding to exotoxins, GL-DVs have demonstrated strong antimicrobial properties and are utilized in creating visible wound dressings through a chromogenic reaction, boosting their potential for clinical use [56].

#### *Lactobacillus rhamnosus GG* (LGG) and *Lactobacillus reuteri* (*L. reuteri*)

A variety of bacteria from *Lactobacilli* spp. have been used in the treatment of periodontitis, such as LGG and *L. reuteri* [216, 217]. GG-DVs have been reported to be preferentially absorbed by LGG in mice gut, and in vitro experiments have shown that these vesicles can directly promote LGG growth. In contrast to the effects of GF-DVs, the contained RNAs that can enhance LGG-mediated inhibition of colitis in mice through activation of the AHR pathway-induced IL-22 expression [154]. Liu et al.. showed that Tartary Buckwheat-derived vesicles also promote LGG growth, and their inclusion of Novel 1, miR-3630, and miR-482b regulates DNA replication, chromosome segregation and other processes in LGGs [155]. Owing to its antimicrobial activities (production of antimicrobial organic acids, ethanol, and reuterin), *L. reuteri* can inhibit the colonization of pathogenic microbes in its vicinity and even reshape the microbial communities in the host [218]. *Portulaca oleracea*-derived vesicles (PO-DVs) have been shown to increase the abundance of *L. reuteri in vivo* and in vitro [156].

### Remodel the immune microenvironment

The periodontal immune microenvironment involves a variety of host immune cells, including neutrophils, macrophages, T cells, B cells, dendritic cells (DCs), and mesenchymal stem cells [219]. The latest opinion is that periodontitis is closely related not only to the pathogenic microbiota, but also to the imbalance of the periodontal immune microenvironment following bacterial infection of the periodontal tissues, leading to periodontitis and tissue destruction [219]. The use of PDVs for periodontitis treatment requires attention not only to the control of the source of infection, but also to the remodeling of the homeostasis of the host immune microenvironment.

#### Macrophages

As the crucial part of the innate immune system, macrophages identify and engulf pathogens and dying neutrophils, and generate specialized pro-resolving mediators, which significantly contribute to the upkeep of homeostasis in the immune microenvironment [220]. When induced by different factors, macrophages polarize into pro-inflammatory M1-type macrophages and anti-inflammatory M2-type macrophages [221]. During the initial phase of inflammation, M1 macrophages are prevalent and release various pro-inflammatory substances like IL-1β, IL-6, IL-12 and IL-23. And in the final stage of inflammation, M2 macrophages produce TGF-β, IL-10 and IFN-γ, contributing to anti-inflammatory and angiogenesis processes [221]. Therefore, induction of macrophage conversion from M1-type to M2-type to reduce inflammation and promote tissue regeneration should be the main direction of PDV-promoted immunotherapy [222].

The GS-DVs were reported to inhibit M1 macrophage polarization and promote M2 macrophage polarization for the treatment of colitis [159]. Zhao et al.. found that GL-DVs were able to reverse lipopolysaccharide (LPS)/D-galactosamine-induced inhibition of autophagy, inhibit M1 polarization, and promote M2 polarization in hepatic macrophages [160]. Notably, Yan et al.. found that osa-miR-164d in GG-DVs directly targets the 3′-UTR of Table 1, which promotes M2 macrophage polarization by downregulating NF-κB expression. Prepared mimetic PDVs using PC to deliver osa-miR-164d could similarly effectively regulate macrophage polarization [162]. Rgl-exomiR-7972 in fresh *Rehmanniae radix*-derived vesicles was able to alleviate LPS-induced lung inflammation by targeting the GPR161-mediated Hedgehog pathway [163]. *Pueraria lobata*-derived vesicles and TM-DVs have the same role in promoting M2 macrophage polarization [164, 165]. In contrast, CR-DVs promoted M1 macrophage polarization via TNF-α/NF-κB/PU.1 axis, and GS-DVs inhibited M2 macrophage polarization. This suggests that PDVs from different sources exhibit different physiological functions in different microenvironments [11, 122].

#### DCs

DCs are professional antigen-presenting cells that, when immature, have a strong phagocytic capacity and act as ‘sentinels’ to investigate the presence or absence of invading microorganisms. While mature DCs have the unique ability to present preserved antigens in secondary lymphoid organs [223, 224]. Pathogenic microorganisms such as *P. gingivalis* can invade DCs and disrupt DC maturation. Maturation molecules like MHCII, CD80/CD86 and CD40, along with inflammatory cytokines such as IL-1b, TNF-α and IL-6, play crucial roles in antigen presentation and the proliferation of effector T cells, including Th17 cells [225]. AY-DVs inhibited LPS-induced phenotypic maturation by reducing the expression of surface molecules in DCs, enhancing the internalization of extracellular antigens, and inhibiting the antigen-presenting capacity of DCs. They also impede the functional maturation of DCs through reducing the expression of both pro-inflammatory and anti-inflammatory cytokines [107]. There have been studies focusing on inhibiting local inflammatory infiltration and ultimately attenuating bone resorption in periodontitis models by inhibiting DC maturation [226]. Furthermore, Deng et al.. showed that broccoli-derived vesicles can attenuate local inflammatory infiltrates by activating the AMPK signaling pathway so that DCs can prevent DCs activation and induce DCs tolerance [167].

#### CD8+/γδ cells

In periodontal tissues, multiple subtypes of T cells play roles in regulating periodontal homeostasis, e.g., Treg cells produce IL-10 and TGF-β to maintain periodontal homeostasis; γδ T cells play roles in barrier monitoring, tissue homeostasis and epithelial repair; and CD8 + T cells downregulate inflammation and inhibit osteoclastogenesis [227]. A study showed that PO-DVs enhanced the abundance of *Lactobacillus reuteri* and increased the levels of indole derivatives, leading to the activation of the aryl hydrocarbon receptor (AhR) and the subsequent downregulation of Zbtb7b, a transcription factor for CD4 + T cells differentiation. Tihs promoted double-positive CD4 + CD8 + T cells expansion to alleviate dextran sulfate sodium-induced colitis [156]. Additionally, GL-DVs were shown to induce endogenous expansion and activation of γδ T cells, yet the exact mechanism still needs to be further explored [168].

### Regulate inflammatory response

As mentioned earlier, dysregulation of the oral microecology and periodontal immune microenvironment will lead to local immune destruction and excessive inflammatory response. Control of the inflammatory response is also one of the main components of periodontitis treatment [151]. Fortunately, several PDVs are known for their powerful anti-inflammatory characteristics.

#### GG-DVs

GG-DVs is known for its strong anti-inflammatory properties and has been linked to the treatment of various inflammatory conditions [228]. Zhang et al.. found GG-DVs can reduce pro-inflammatory cytokines (TNF-α, IL-6, IL-1β) and boost anti-inflammatory cytokines (IL-10 and IL-22), as well as promote the proliferation of intestinal epithelial cells (IECs) to enhance intestinal repair in a mouse model of colitis [169]. Additionally, GG-DVs lipids were proven to reduce the activity of NLRP3 inflammasomes in various macrophages [140]. According to Yin et al.., the miRNA profile of GG-DVs was characterized, showing that they reduced the expression of NF-κB and multiple inflammatory cytokines in the LPS-induced inflammatory response in intestinal cells [170]. These suggest that GG-DVs provide a natural means of transferring bioactive materials to the human gut, thus regulating intestinal inflammation [170].

#### GL-DVs

GL-DVs possess strong anti-inflammatory effects and are utilized in treating inflammation of the intestines and liver. A study shows that han-miR-3630-5p in GL-DVs can alleviate colitis in mice by directly binding to the 3’UTR region of TLR4, and then regulating the TLR4/MyD88/NF-κB pathway [158]. GL-DVs are also used in liver diseases, e.g., Zhao et al.. demonstrated that GL-DVs promote autophagy in liver macrophages, leading to the inhibition of NLRP3 inflammasome activation, and act as hepatoprotective agents to alleviate acute liver failure [160]. GL-DVs have also been found to dose-dependently reduce the expression of inflammatory factors in THP-1 macrophages, while having no significant effect on primary cultured Kupffer cells [172].

#### LM-DVs

Previous studies have shown that LM-DVs can achieve anti-inflammatory properties in LPS-induced mouse macrophages by inhibiting the ERK1/2-NF-κB signaling pathway. In addition, they validated the anti-inflammatory properties of LM-DVs in CD4, CD8 and γδ T cells [176]. Tinnirello et al.. showed that industrially produced LM-DVs can ameliorate experimental colitis-associated injuries in rats through anti-inflammatory and antioxidant responses, suggesting that existing research is taking a big step towards industrial generation and large-scale application of PDVs [175].

#### Other PDVs

MiR-146a, miR-146b, miR-125a and let-7e in apple-derived vesicles (AP-DVs) work at different stages of inflammation to inhibit the NF-κB pathway and alleviate the inflammatory response [173]. AP-DVs have also been shown to function in fibroblasts, suggesting that they can produce anti-inflammatory signals in multiple cell lines [173, 174]. Moreover, MC-DVs, GC-DVs and Shiitake Mushroom-derived vesicles were all shown to exert anti-inflammatory effects by inhibiting the NLRP3 inflammasome [58, 124, 177].

### Regulate oxidative stress

In the past few years, increasing research has examined the impact of oxidative stress on periodontitis, and defined periodontitis as an inflammatory disease of oxidative stress [229, 230]. Oxidative stress is characterized by an imbalance between excess ROS and antioxidants [231, 232]. ROS can be considered as a ‘double-edged sword’. On the one hand, ROS play an important role in cell signaling, gene regulation and antimicrobial defense. On the other hand, excessive ROS cause protein oxidation, lipid peroxidation, enzyme inhibition, etc., which can lead to periodontal destruction [229]. Under physiological conditions, the body produces a range of antioxidants to maintain the balance with ROS, such as superoxide dismutase (SOD), catalase (CAT), glutathione peroxidase (GSH-PX), glutathione reductase, and DNA repair enzymes [232]. In the case of periodontitis, more and more studies are using antioxidants to reverse the damage caused by oxidative stress [233]. Due to the inherent antioxidant properties of many plants, researchers have used PDVs as a good antioxidant [234].

The antioxidant response in cells is significantly influenced by the transcription factor Nrf2 [235]. Under stress, Nrf2 can translocate to the nucleus and thus upregulate the expression of antioxidant genes [236]. Previous studies have shown that 6-shogaol from GG-DVs significantly increased nuclear translocation of Nrf2 in hepatocytes, thereby reducing ROS production [184]. Zhao et al.. found that blueberry-derived vesicles (BB-DVs) reversed the effects of rotenone in reducing Nrf2 levels in the nucleus of HepG2 cells, and accelerated nuclear translocation of Nrf2 [185]. Another study found that LR-DVs attenuated β-amyloid (Aβ)-induced oxidative stress in HT22 cells by promoting nuclear translocation of Nrf2 and upregulating HO-1 and NQO1 expression [186]. In addition, GG-DVs, AV-DVs and TMT-DVs also play a role in activating the Nrf2 pathway to counteract oxidative stress [106, 144, 183].

In addition, some PDVs can also directly upregulate antioxidant levels. Wang et al.. found that MC-DVs upregulated the levels of GSH-PX, SOD, and CAT, which may be related to thioredoxin and peroxidase in the vesicles [188]. Another study used hybrid vesicles from grapes and tomatoes to enhance the enzymatic activities of SOD, CAT and GSH-PX, resulting in synergistic antioxidant effects [189]. Notably, *Robinia pseudoacacia* -derived vesicles (RP-DVs) downregulated the expression of NOX4 and ALOX5, which drive ROS production and lipid peroxidation, respectively [190].

### Promote periodontal tissue regeneration

Periodontal tissue regeneration is a complex and highly specialized process of reconstruction of tooth-supporting tissues, including regeneration of both hard (alveolar bone and cementum) and soft (gingiva and periodontal ligament) tissues [237]. For hard tissue regeneration, it has been concentrated on inducing bone regeneration by inhibiting osteoclast differentiation and promoting osteoblast proliferation and differentiation. However, there are no studies that have used PDVs for cementum regeneration. The gingival and periodontal ligaments are complex anatomical structures. For example, the periodontal ligament has an extensive blood supply, a nerve network and multiple cell populations such as fibroblasts, periodontal ligament stem cells, endothelial cells and nerve cells [238]. Soft tissue regeneration is therefore concentrated on promoting angiogenesis, neural differentiation, and migration of fibroblasts and keratinocytes.

#### Reconstruction of periodontal bone homeostasis

Local formation of osteoclasts and their stimulation is necessary for alveolar bone loss. A variety of mediators, such as IL-1, IL-6, IL-11, IL-17, TNF-α, TNF-β, TGF-β, kinins and thrombin can stimulate bone resorption [239]. Seo et al.. demonstrated that GS-DVs can induce the inhibition of RANKL-induced osteoclast differentiation of bone marrow-derived macrophages, reduction of osteoclast numbers and thus inhibition of bone loss in an LPS-induced calvaria loss model in mice [204]. In vitro experiments demonstrated that AP-DVs promote osteoblastogenesis in osteoblastic MC3T3-E1 cells by modulating the BMP2/Smad1 pathway [207]. Hwang et al.. demonstrated that yam-derived vesicles (Yam-DVs) may induce osteoblast differentiation through the BMP-2/p-p38-dependent Runx2 pathway, and promote longitudinal bone growth and mineral density of the tibia in ovariectomized osteoporotic mice [206]. Plum-derived vesicles have been shown to play a role in the balance between bone formation and bone resorption. They can promote osteogenic differentiation of MC3T3-E1 cells through the BMP-2/MAPK/Smad-1-dependent Runx2 pathway, and also inhibit osteoblastic differentiation of primary mouse osteoclasts [205].

#### Angiogenesis and soft tissue regeneration

Angiogenesis is important for maintaining healthy periodontal tissues, and is also considered a component of periodontal regernation [240]. Xu et al.. reported that GS-DVs significantly increased the levels of angiogenesis-related factors, such as VEGF and HIF-1α, in rat wound-healing tissues. Also, GS-DVs induced neural differentiation of bone marrow mesenchymal stem cells by upregulating PI3K signaling [143]. In addition, GF-DVs, wheat-derived vesicles (WT-DVs) and *Aloe saponaria*-derived vesicles enhanced tube formation capacity of human umbilical vein endothelial cells (HUVECs), although the exact mechanism is unclear [200, 202, 203].

Despite the variability between intraoral fibroblasts (periodontal ligament, gingival and oral mucosal fibroblasts) and dermal fibroblasts (HDFs), it cannot be denied that PDVs has a borrowed role for intraoral fibroblasts in promoting the functions of HDFs [241]. Kim et al.. reported that AS-DVs increased the proliferation and migration of HDFs in a dose-dependent manner [200]. Opuntia ficus indica-derived vesicles have also been shown to promote the migration of HDFs as potential candidates for healing chronic skin wounds [119]. Epithelial tissue provides a barrier between the body and the environment, and the keratinocytes form the first line of defense against bacterial attack [242]. Studies indicated that both AV-DVs and WT-DVs promoted the migration of HaCaT cells (i.e., Spontaneously immortalized human keratinocytes). However, the specific mechanism of it still needs to be further explored [106, 203].

## PDVs and periodontitis-associated systemic disease treatment

**Fig. 7:**
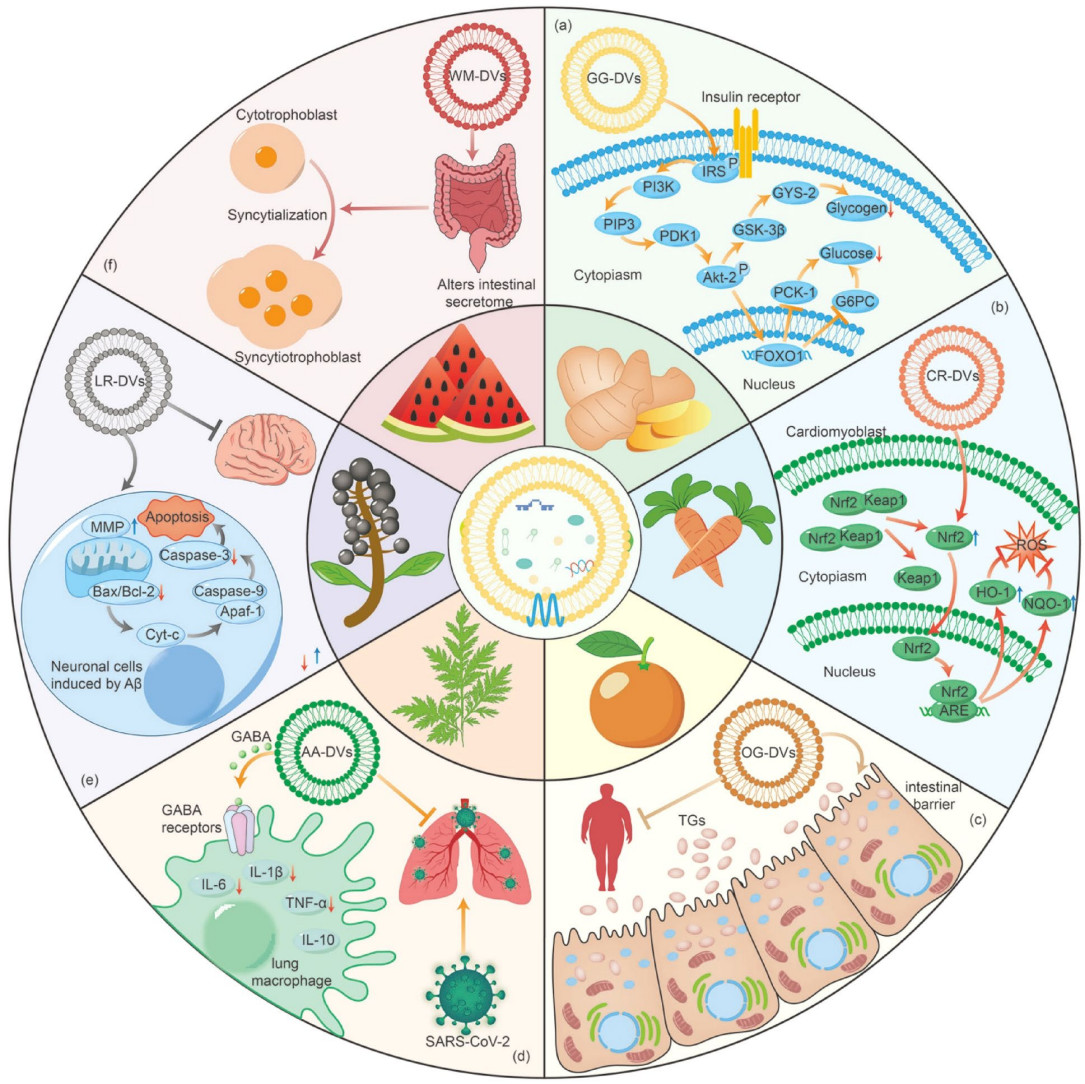
PDVs for the treatment of periodontitis-associated systemic diseases. (**A**) Type II diabetes mellitus; (**B**) Cardiovascular diseases; (**C**) Obesity; (**D**) COVID-19; (**E**) Alzheimer’s disease; (**F**) Adverse pregnancy

A consensus report published by the European Federation of Periodontology and WONCA Europe states that periodontitis is associated with a variety of systemic diseases such as diabetes, cardiovascular diseases, COVID, etc [243]. Periodontitis can affect systemic diseases through mechanisms such as blood circulation, the oral-gut axis, and inflammation [244–246]. Compared to mammalian-derived vesicles, PDVs not only have similar therapeutic effects by inheriting the bioactive profile of the source plants from which it is derived, but also have low-immunogenicity and wide range of sources for the treatment of a wide range of diseases [247, 248]. In this section, we will discuss the possibilities of PDVs for the treatment of periodontitis-associated systemic diseases **(**Fig. 7; Table 3**)**.

**Table 3:** Application of PDVs in different periodontitis associated systemic diseases

Disease	Plant type	In/ex vivo model sample involved	Main findings	References
Type II Diabetes Mellitus	Garlic	(1) In vitro: BV2 microglial cells (2) In vivo: 6–8 weeks C57BL/6 mice	Train human gut Akkermansia muciniphila can reverse high-fat diet-induced T2DM in mice	[249]
	Tangerine peel	(1) In vitro: VoLo and AML-12 Cells (2) In vivo: 7 weeks male C57BL/Ks mice and C57BL/KsJ-*db/db* mice	Improve glucose and lipid metabolism via activating the expression of genes related to fatty acid β-oxidation and glycolysis	[250]
	*Aloe vera*, *Azadirachta indica* and *ginger*	(1) In vivo: Wistar male rats	Treat diabetic wounds by oxygenating and reducing inflammation	[251]
	Mung bean sprouts	(1) In vitro: AML12 and HepG2 cell lines (2) In vivo: 8 weeks male C57BL/6 mice	Upregulated GLUT4 & Nrf2 and downregulated GSK-3β via activating the PI3K/Akt signaling pathway, promoting the production of antioxidant enzymes, such as HO-1 and SOD, to reduce oxidative stress	[252]
	Turmeric	(1) In vitro: Mouse fibroblast L929 and mouse RAW264.7 (2) In vivo: 6–8 weeks male C57BL/6J mice	Restoration of the fibroblast-macrophage communication network stimulates cell regeneration and thus promotes diabetic wound healing.	[146]
	Ginger	(1) In vivo: 7 weeks male C57BL/6J mice	Improve insulin resistance and islet β-cell function	[15]
	Ginseng	(1) In vitro: Human umbilical vein endothelial cells (HUVECs) (2) In vivo: 8–12 weeks male B6.BKS(D)-Lepr^db^/J (db/db) mice	Stimulate glycolysis reprogramming-mediated angiogenesis in diabetic ulcers	[253]
	Ginger	(1) In vitro: insulin resistant HepG2 cells (2) In vivo: 6 weeks male C57BL/6J mice (T2DM Mice Model)	Improve glucose and fatty acid metabolism and protect pancreatic β-Cells in T2DM mice	[254]
Cardiovascular Diseases	Grapefruit	(1) In vitro: primary mouse vascular smooth muscle cells (2) In vivo: 6 − 8 weeks male C57BL/6 mice	Drivie M2 macrophage polarization, reduce inflammation, and suppress bone-vascular axis	[118]
	Avocado	(1) In vitro: mice peritoneal macrophages	Suppress activation of NFκB and NLRP3, and inhibit expression of pro-inflammatory and atherogenic genes	[108]
	Carrot	(1) In vitro: human neuroblastoma SHSY5Y cells, embryonic rat heart-derived cardiomyoblasts (H9C2)	Inhibit ROS generation and apoptosis induction	[187]
	*Momordica charantia*	(1) In vitro: rat cardiomyocyte cell (H9C2) (2) In vivo: 5–6 weeks BALB/c nude mice	Scavenge elevated mitochondria ROS	[83]
	*Carthamus tinctorius*	(1) In vitro: human umbilical vein endothelial cells (2) In vivo: 8 weeks male C57BL/6 mice	Anti-inflammatory effect by miR166a-3p/CXCL12 pathway	[120]
Obesity	Turmeric	(1) In vitro: mouse embryonic fibroblasts (3T3-L1) (2) In vivo: male C57BL/6J mice	Activate TRPV1, leading to an increased calcium influx that initiated a cascade of metabolic pathways, ultimately enhancing lipolysis, inhibiting lipogenesis, promoting adipocyte browning, and triggering apoptosis	[45]
	*Brassica*	(1) In vitro: dynamic gastrointestinal model	The microbiome was scarcely affected by the treatments in terms of microbiota composition or the *Bacteroidetes/Firmicutes* ratio	[255]
	Orange	(1) In vitro: human colorectal carcinoma cells (ATCC HTB-37) (2) In vivo: 4 weeks male mice	Induce the release of chylomicron-associated triglycerides and modulated the expressions of Genes involved in lipid absorption and release in jejunum and liver	[43]
	Spinach	(1) In vitro: mouse embryonic fibroblasts (3T3-L1) (2) in vivo: 4 weeks C57BL/6J mice	Suppress lipid accumulation during adipocyte differentiation and reduce adipose tissue weight and body weight gain by downregulating key adipogenic transcription factors	[256]
	Kidney beans	(1) In vivo: Waster male rats	Increase the diversity of gut microbiota, and improve obesity by regulating the composition of gut microbiota, increasing the distribution of beneficial bacteria and enhancing the level of metabolite SCFAs	[257]
	Garlic	(1) In vitro: BV2 microglia cells (2) In vivo: 10–12 weeks WT C57BL/6, IDO1^−/−^ and AHR^−/−^male mice	Reverse high-fat diet induced obesity via the gut/brain axis	[16]
	Garlic	(1) In vitro: mouse-derived RAW264.7 macrophages and 3T3-L1 cells (2) In vivo: 6–8 weeks C57BL/6 mice	Carry miR-396e shapes macrophage metabolic reprograming to mitigate the inflammatory response in obese adipose tissue	[258]
COVID-19	*Artemisia annua*	(1) In vitro: Mouse alveolar macrophages (MH-S), Human embryonic kidney cells (293T) and Madin-Darby canine kidney (MDCK) cells (2) In vivo: 6–8 weeks C57BL/6 male mice	Alleviate lung immunopathology and raise the survival rate of challenged mice	[44]
	Ginger	(1) In vitro: mouse C57BL/6 lung carcinoma LLC1 and macrophage cell lines, monkey kidney Vero E6 cells, and human alveolar basal epithelial A549 and monocytic U937 cell lines (2) In vivo: 8–12 weeks C57BL/6 male mice	Inhibit Nsp12 and spike gene expression.	[171]
Alzheimer’s disease	*Citrus limon*	(1) In vitro: human neuroblastoma (SH-SY5Y cell line)	Antioxidant properties and blood–brain barrier permeability	[259]
	*Lycium ruthenicum*	(1) In vitro: mouse hippocampal neuronal cells (HT22 cells)	Alleviated Aβ-induced oxidative stress and apoptosis	[186]
	*Lycium ruthenicum*	(1) In vitro: PC12 cells	Content ata-miR156c-3p mitigates Aβ-induced cytotoxicity in PC12 cells	[46]
Adverse pregnancy	Watermelon	(1) In vitro: Caco-2 cells and BeWo cells	Internalize by human intestinal cells to alter the intestinal cell secretome which promotes trophoblast migration and fusion	[260]

### T2DM

Diabetes and periodontitis may show bidirectional correlation through mechanisms such as inflammatory response, oxidative stress and insulin resistance [261, 262]. Currently, T2DM is treated with medication in conjunction with exercise and diet [263]. However, several meta-analyses have confirmed that drug-related side effects cannot be ignored [264, 265]. Recent studies have confirmed that mung bean sprout-derived vesicles can increase the number of islet B cells and reduce insulin resistance in mice to alleviate T2DM symptoms [252]. Recent studies have shown that GG-DVs can reduce fasting blood glucose levels and improve glucose tolerance in T2DM mice as effectively as metformin. Further studies showed that miRNAs in vesicles regulate the phosphatidylinositol 3-kinase (PI3K)/Akt-2 signaling pathway and exhibit strong interactions with various target mRNAs responsible for hepatic gluconeogenesis, ectopic fat deposition and oxidative stress [254]. In addition to the direct pathways, there has also been an innovative use of GL-DVs to train gut bacteria and reverse insulin resistance, suggesting a new pathway for the potential application of PDVs [249].

In addition, PDVs can also be used to treat diabetic complications. Zou et al.. showed that tangerine peel-derived vesicles attenuated T2DM-induced hepatic steatosis by ameliorating insulin resistance, modulating lipid metabolism and gut flora [250]. Vesicles from *Aloe vera s*, *Azadirachta indica* and *ginger* help to reduce oxidative stress to promote diabetic wound healing [251]. Furthermore, it has also been investigated that *Citrus limon* source vesicles are packed into hydrogels constructed from gelatin with methacryloylmethacrylate (GelMA) and dialdehyde starch (DAS), providing excellent performance for diabetic wound healing [266].

### Cardiovascular diseases

Several meta-analyses have shown that patients with periodontitis have a higher risk of developing cardiovascular diseases such as carotid artery calcification and coronary heart disease [267, 268]. Periodontitis may induce cardiovascular diseases through mechanisms such as triggering a systemic inflammatory response [246], damage to cellular components by oxidative stress due to ROS accumulation [269] and damage to the vascular endothelium by periodontal pathogens that enter the circulation [270].

It has been suggested that atherosclerosis is an inflammatory disease rather than simply a cholesterol storage disease. Macrophages play key roles in both early atherogenesis and advanced plaque progression [271, 272]. The latest study showed that avocado-derived vesicles (AC-DVs) loaded with ginkgetin and berberine own strong anti-inflammatory effects. They can be applied to reduce the activation of NFκB and NLRP3, and inhibit foam cell production in macrophages for the treatment of atherosclerosis [108]. It has also been shown that PDVs not only act as transport carriers, but also exert anti-inflammatory effects themselves, e.g. GF-DVs were significantly associated with atherosclerosis-related signaling pathways [118].

The accumulation of excess ROS leads to oxidative stress, which directly or indirectly induces gene mutations, protein denaturation, and lipid peroxidation. Therefore, ROS are considered to be an important risk factor for cardiovascular disease [273]. Preliminary studies have shown that carrot-derived vesicles acting on cardiomyoblasts significantly inhibit ROS production and apoptosis [187]; similarly, MC-DVs are able to scavenge elevated ROS in cardiomyocytes and balance the mitochondrial membrane potential, thereby protecting cardiomyocytes [83]. Further, *Carthamus tinctorius*-derived vesicles were demonstrated to show high efficacy in reducing apoptosis and oxidative stress in human venous endothelial cells, which is expected to serve as a therapeutic agent for atherosclerosis [120].

Although it has been shown that periodontal pathogens such as *Tannerella* and *Anaeroglobus* are not only associated with periodontitis but are also enriched in patients with carotid atherosclerosis [274], there are no studies on the treatment of cardiovascular disease by targeting periodontal pathogens through PDVs.

### Obesity

The complex link between obesity and periodontitis has become an important focus in periodontal medicine research [275]. Many epidemiological investigations have revealed a link between the two diseases [276, 277]. The use of anti-obesity drugs is currently an important measure in the treatment of obesity. However, its widespread use has received limitations in terms of adverse effects [278]. Therefore, some researchers have focused on using PDVs to treat obesity by regulating lipid metabolism and modulating gut flora.

With regard to the regulation of lipid metabolism by PDVs, Berger et al.. showed that OG-DVs promote the release of chylomicron-associated triglycerides, and downregulate mRNA coding for MTP, which have a major role in chylomicron synthesis [43]. At the transcriptional level, SN-DVs regulate lipid metabolism by inhibiting the expression of key transcription factors involved in lipid synthesis, namely PPAR-γ and C/EBP-α, highlighting its crucial role in preventing adipogenesis [256].

Numerous previous studies used plant extracts to improve obesity by remodeling gut flora, with different plant extracts exerting different effects [279, 280]. Similarly, whether PDVs play a role in modulating the gut flora seems to be influenced by the plant source. Wang et al.. showed that TM-DVs significantly enhanced lipolysis, inhibited lipogenesis and altered the composition of intestinal flora and effectively reduced the *Firmicutes*/*Bacteroidota* (F/B) ratio, a recognized biomarker of obesity-related dysbiosis [45, 281]. In addition, kidney bean-derived vesicles may also alleviate obesity symptoms by increasing short-chain fatty acids and the diversity of gut flora [257]. However, *Brassica*-derived vesicles loaded with red cabbage extracts were used to determine the flora in a dynamic gastrointestinal model, where the microbial composition and the F/B ratio were scarcely affected [255].

### COVID-19

The typical clinical feature of COVID-19 is diffuse alveolar damage that leads to acute respiratory distress syndromes [282], and a significant association of its complications with periodontitis has been revealed [283]. Researchers selected 260 miRNAs from 13 types of PDVs for target site prediction against the SARS-CoV-2 genomic sequence. The results showed that 11 miRNAs had absolute target specificity towards SARS-CoV-2 [284]. Further experiments demonstrated that *Artemisia*-derived vesicles (AM-DVs) were able to reduce oxidative stress and inflammation, thereby alleviating SARS-CoV-2 pseudovirus-induced lung injury [44]. Notably, ary-miR-396a-5p- and rlcv-miR-rL1-28-3p in GG-DVs inhibited the expression of Nsp12 and spike genes, respectively. They were used to alleviate SARS-CoV-2-induced lung inflammation [171].

The development of a safe and effective vaccine is an important way to prevent COVID-19 [285]. Compared to other synthetic delivery systems (e.g., lipid nanoparticles, adenovirus), PDVs have distinct advantages such as good biocompatibility and being natural products with safety [286]. Therefore, researchers are focusing on delivering RNA vaccines via PDVs. Pomatto et al.. demonstrated that mRNA from SARS-CoV-2 can be loaded into OG-DVs. The complex can be delivered to target cells to activate lymphocyte responses. The efficacy of oral administration and intranasal administration were also validated [287]. Further studies showed that PDVs loaded with SARS-CoV-2 S1 mRNA were able to be preserved for long periods of time in edible gastro-resistant capsules and oral administration resulted in effective vaccination [288]. It is notable that PDVs is not only suitable for the delivery of mRNA, but may also be suitable for the delivery of other nucleic acid molecules, such as siRNA, miRNA and DNA [289].

### Alzheimer’s disease (AD)

Periodontitis and periodontal pathogens are the real risk factors for the onset or worsening of AD [290, 291]. AD is associated with abnormal deposits of proteins in the brain, specifically Aβ plaques and tau tangles [292]. Compared to the application of mammal-derived vesicles in the treatment of AD, the use of PDVs for treating AD is still in its early stages [293]. Experiments indicated that LM-DVs have blood-brain barrier permeability and can improve oxidative stress in SH-SY5Y cells (human neuroblastoma) induced by Aβ [259]. Zhang et al.. focused on the potential applications of LR-DVs in AD [46, 186]. They found that LR-DVs can mitigate Aβ-induced apoptosis in HT22 cells (mouse hippocampal neuronal cells) by enhancing mitochondrial membrane potential, decreasing Bax/Bcl-2 ratio, reducing cleaved Caspase-3 expression, promoting the nuclear translocation of Nrf2, and upregulating the expression of HO-1 and NQO1 [186]. Furthermore, the inhibition of apoptosis may be associated with the MAPK and PI3K/Akt signaling pathways [46].

### Adverse pregnancy

Periodontitis is strongly associated with a variety of adverse pregnancy outcomes (e.g., preterm labour, low birth weight) [294, 295]. There are few studies on PDVs to improve adverse pregnancies. Only one study has shown that watermelon-derived vesicles are taken up by intestinal epithelial cells to alter the basal secretome, thereby enhancing two important processes for efficient placental function: trophoblast invasion and syncytialisation, and also potentially ameliorating fetal growth restriction [260]. Previous studies have shown that many plant extracts (e.g. taxifolin and resveratrol) play anti-inflammatory and anti-oxidative stress roles in human embryonic trophoblasts to prevent adverse pregnancy outcomes [296, 297]. Furthermore, some NPDVs can enter the human placenta [298]. Therefore, it is promising to take advantage of the low toxicity and low immunogenicity of PDVs to develop more drugs with the potential to treat adverse pregnancies.

## Challenges and prospects

### Kill two birds with one stone

**Fig. 8:**
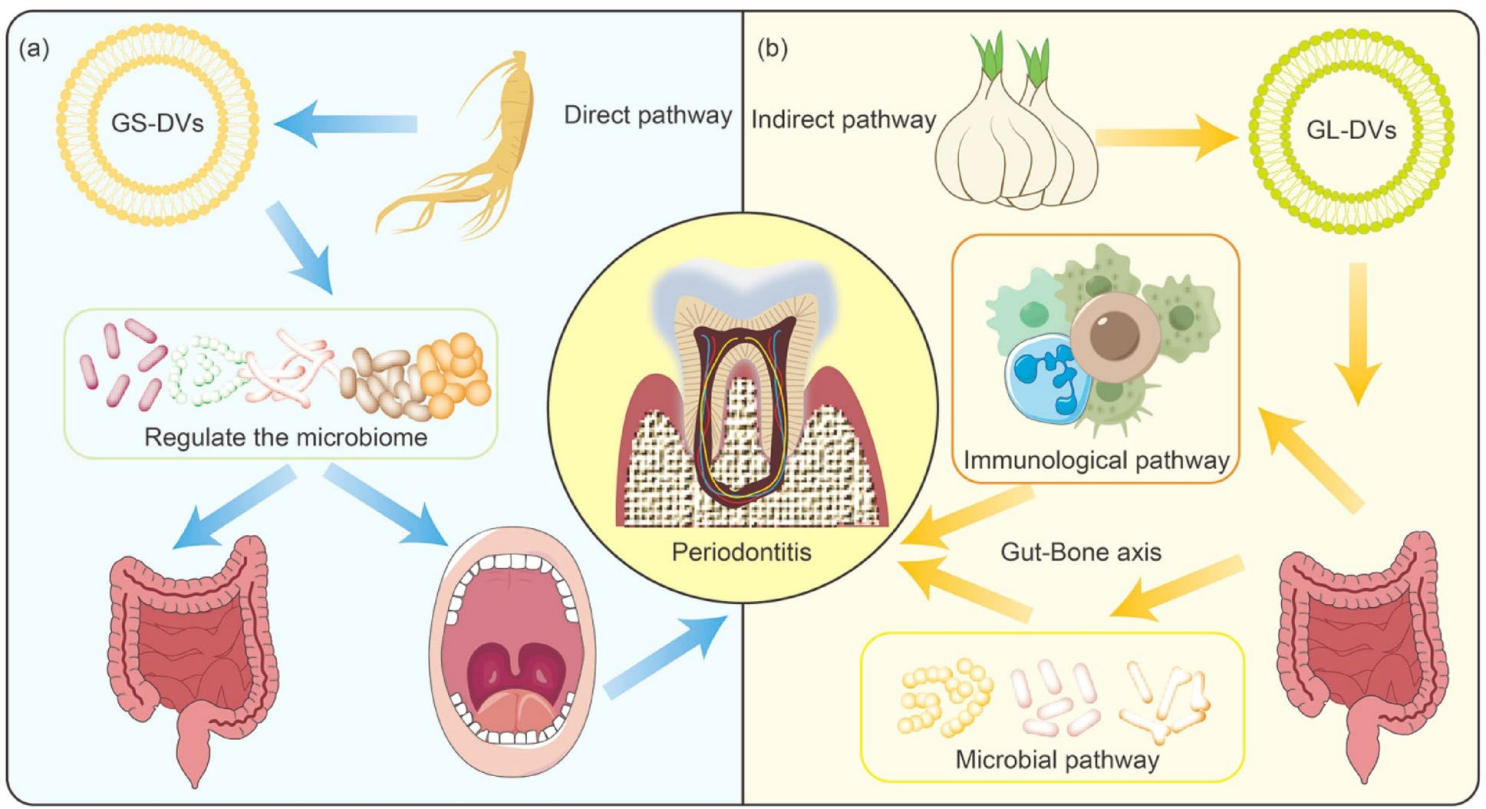
Kill two birds with one stone. (**A**) direct pathway: GS-DVs treat both gut disease and periodontitis by regulating the microbiome of the oral cavity and gut; (**B**) indirect pathway: GL-DVs alleviate periodontitis through the immunological and microbial pathways of the gut-bone axis

Taking into account the potential linkages between different diseases, the strategy of ‘killing two birds with one stone’ will bring greater benefits to patients by addressing multiple problems at the same time and improving treatment outcomes **(**Fig. 8**)**. The link between periodontitis and gut inflammation might be established through microbial pathway and immunological pathway, also known as the oral-gut axis [299, 300]. For example, GS-DVs play a role in suppressing inflammation and improving the progression of colitis [159]. GL-DVs, on the other hand, inhibit colitis progression by modulating the gut microbiota [158]. Therefore, we hypothesize that oral PDVs is promising for the simultaneous treatment of periodontitis and gut inflammation.

There is growing evidence for the role of the gut-bone axis in periodontitis. Gut pathogens or metabolites may be translocated to distant alveolar bone via the circulation to modulate bone homeostasis. Gut microbial translocation also enhances systemic inflammation, which may exacerbate periodontitis [301, 302]. Current evidence suggests that PDVs plays an important role in remodeling gut microbial homeostasis [257]. A study suggests that GL-DVs enriched with peu-miR-2916-p3 can remodel dextran sulfate sodium (DSS)-induced gut microbial dysbiosis, promising therapeutic use in colitis [303]. Thus, PDVs is expected to prevent or treat periodontitis by modulating the gut-bone axis in the treatment of digestive disorders.

### Hybrid PDVs

In future studies, the development of hybrid vesicles will provide a new perspective and possibility for the application expansion of PDVs. The design of hybrid vesicles through membrane fusion between different types of vesicles, as an emerging technology, can not only overcome the limitations of single vesicles in terms of targeting, stability and drug loading efficiency, but also significantly enhance the efficiency of cellular uptake [189, 304]. This hybridization strategy can not only achieve effective integration of PDVs with similar functions, enabling them to perform more powerful functions than a single PDVs [305]. In addition, synergistic effects can also be exerted through the fusion of different functional vesicles, thus enhancing their overall performance and application potential [26]. For example, Lu et al.. used *Exocarpium Citri grandis*-derived vesicles with anti-inflammatory and antioxidant properties to hybridize with MSC membrane-derived vesicles with macrophage-targeting capacity to address inflammatory macrophage-mediated rejection in cardiac transplants, demonstrating the great potential of hybrid vesicles in clinical applications [306]. Future research should focus on the design of hybrid vesicles capable of carrying multiple drugs or bioactive molecules at the same time for use in combination therapy [307]. Furthermore, the development of standardized methods for the preparation and characterization of hybrid vesicles is essential for their successful implementation.

### Stability of PDVs

One of the key challenges in applying PDVs is their stability under different storage conditions. In terms of storage temperature, BB-DVs were stored at −80 °C, −20 °C, 4 °C, and 25 °C for 7 and 30 days, respectively. It was found that storage at 25° tended to lead to microbial growth, with the smallest size change in the 4 °C group for short-term storage (7 days) and the smallest change in the − 80 °C group for long-term storage (30 days) [308]. Another study showed that the free fatty acid content of vesicles of *Kaempferia parviflora*-derived vesicles was maintained from 0 to 8 weeks at −20 °C and − 80 °C, while slowly degraded at 4 °C [309]. Thus, PDVs should be stored at −80 °C as much as possible. Interestingly, the size and yield of olive-derived vesicles remained unchanged after exposure to high temperatures (70 °C for 1 h), a wide pH range (5–10), and 50–100 nm extrusion, demonstrating high resistance to physical and chemical stresses [310]. The stability of PDVs is also related to the isolation method, Jiang et al.. used a combination of ultracentrifugation and ExoQuick system to isolate GS-DVs with higher stability than a single method [311]. Different preservatives should also be taken into account. PDVs with TMO as a preservative were more stable compared to the group without preservatives or the group with 1,3-butylene glycol [312]. TMO consists of an extract of *Illicium verum*, caprylyl glycol, 1,2-hexanediol, and butylene glycol [312]. In the future, developing new storage media to improve the stability of PDVs also needs further exploration [313].

## Conclusion

PDVs are used as a promising therapeutic tool for the treatment of periodontitis and its associated systemic diseases. PDVs are expected to treat periodontitis by maintaining oral microecological homeostasis, remodeling the immune microenvironment, regulating the inflammatory response and oxidative stress, as well as promoting periodontal tissue regeneration. In addition, PDVs have shown encouraging results in the treatment of systemic diseases associated with periodontitis. Despite significant progress in understanding the role of PDVs in disease treatment, some challenges remain, such as unifying confusing nomenclature and optimizing storage conditions. Further research is needed to address current challenges and realize the full therapeutic potential of PDVs. As the field continues to advance, PDVs is expected to become a promising treatment modality.

## Data Availability

No datasets were generated or analysed during the current study.

## Abbreviations

PDVs: Plant-derived vesicles
T2DM: Type II diabetes mellitus
EVs: Extracellular vesicles
MVBs: Multivesicular vesicles
MISEV: Minimal information for studies of extracellular vesicles
PDEVs: Plant-derived extracellular vesicles
PDEVMs: Plant-derived extracellular vesicles mimetics
PDACDVs: Plant-derived artificial cell-derived vesicles
PDSVs: Plant-derived synthetic vesicles
EXPO: Exocyst-positive organelle
NPDVs: Non-plant-derived vesicles
dUC: Differential ultracentrifugation
DGC: Density gradient centrifugation
PBP: PEG-based precipitation
UF: Ultrafiltration
IAC: Immuno-affinity capture
EP: Electrophoresis
TFF: Tangential flow filtration
*F. nucleatum*: *Fusobacterium nucleatum*
*P. gingivalis*: *Porphyromonas gingivalis*
*S. aureus*: *Staphylococcus aureus*
LGG: *Lactobacillus rhamnosus GG*
*L. reuteri*: *Lactobacillus reuteri*
AD: Alzheimer’s disease
